# *Lotus tenuis* x *L. corniculatus* interspecific hybridization as a means to breed bloat-safe pastures and gain insight into the genetic control of proanthocyanidin biosynthesis in legumes

**DOI:** 10.1186/1471-2229-14-40

**Published:** 2014-02-03

**Authors:** Francisco J Escaray, Valentina Passeri, Florencia M Babuin, Francisco Marco, Pedro Carrasco, Francesco Damiani, Fernando L Pieckenstain, Francesco Paolocci, Oscar A Ruiz

**Affiliations:** 1 IIB-INTECH/CONICET-UNSAM, Chascomús, Bs. As, Argentina; 2 National Research Council, Institute of Biosciences and BioResources -Perugia (CNR-IBBR), Perugia, Italy; 3 Department of Plant Biology, Universitat de València, València, Spain; 4 Department of Biochemistry and Molecular Biology, Universitat de València, València, Spain

**Keywords:** Interspecific hybridization, *Lotus*, Proanthocyanidins (PAs), *TT2*, Forage legumes, Nutritive value

## Abstract

**Background:**

Proanthocyanidins (PAs) are secondary metabolites that strongly affect plant quality traits. The concentration and the structure of these metabolites influence the palatability and nutritional value of forage legumes. Hence, modulating PAs in the leaves of forage legumes is of paramount relevance for forage breeders worldwide. The lack of genetic variation in the leaf PA trait within the most important forage species and the difficulties in engineering this pathway via the ectopic expression of regulatory genes, prompted us to pursue alternative strategies to enhance this trait in forage legumes of agronomic interest. The *Lotus* genus includes forage species which accumulate PAs in edible organs and can thus be used as potential donor parents in breeding programs.

**Results:**

We recovered a wild, diploid and PA-rich population of *L. corniculatus* and crossed with *L. tenuis*. The former grows in an alkaline-salty area in Spain while the latter is a diploid species, grown extensively in South American pastures, which does not accumulate PAs in the herbage. The resulting interspecific hybrids displayed several traits of outstanding agronomic relevance such as rhizome production, PA levels in edible tissues sufficient to prevent ruminal bloating (around 5 mg of PAs/g DW), biomass production similar to the cultivated parent and potential for adaptability to marginal lands. We show that PA levels correlate with expression levels of the *R2R3MYB* transcription factor *TT2* and, in turn, with those of the key structural genes of the epicatechin and catechin biosynthetic pathways leading to PA biosynthesis.

**Conclusions:**

The *L. tenuis* x *L. corniculatus* hybrids, reported herein, represent the first example of the introgression of the PA trait in forage legumes to levels known to provide nutritional and health benefits to ruminants. Apart from PAs, the hybrids have additional traits which may prove useful to breed forage legumes with increased persistence and adaptability to marginal conditions. Finally, our study suggests the hybrids and their progeny are an invaluable tool to gain a leap forward in our understanding of the genetic control of PA biosynthesis and tolerance to stresses in legumes.

## Background

Livestock production is the world’s largest use of land resources. This activity occupies 3.4 billion hectares for grazing and 0.5 billion hectares for feed crops, equivalent to 80% of all agricultural lands [1]. Livestock productivity varies widely and largely depends on pastures. Legumes growing in pastures increase both the productivity and nutritional value of forages [2]. Indeed, improving the quality and environmental adaptability of pastures to marginal areas, while decreasing the ecological impact of ruminant livestock are becoming crucial issues to meet the rising world’s demand for cheap and safe livestock food products and for truly sustainable livestock farming. Among the compounds that strongly affect legume quality, proanthocyanidins (PAs), also known as condensed tannins, are of outstanding relevance because the nutritional value of forage legumes is highly influenced by the concentration and structure of these compounds [3]. Moderate quantities of PAs (about 5 mg PAs/g DW) in forage, prevent proteolysis during ensiling and rumen fermentation, thereby protecting ruminants against pasture bloat [4-6]. In fact, by binding to proteins in the rumen, PAs reduce fermentation rate and increase the levels of proteins and essential amino acids passing through the rumen of grazing animals. Therefore, PAs make the conversion of plant protein into animal protein more efficient and reduce the need to include supplemental protein in the diet [7]. Additionally, decreased protein degradation in the rumen diminishes methane production and ammonium excretion in urine. Methane and nitrous oxide from pastures are potent greenhouse gases (GHG); methane produced by ruminants accounts for 17-37% of anthropogenic methane [8,9]. However, high concentrations of PAs are associated with lowered palatability and reduced forage intake [3]. Thus, obtaining legumes with moderate quantities of PAs is one of the priorities of forage breeders worldwide. PAs only accumulate in the seed coats of the most valuable forage species, such as alfalfa (*Medicago sativa*) and clovers (*Trifolium* spp.) but are absent from their leaves [10]. Notably, neither ecotypes nor wild relatives of these legume species accumulate PAs in the leaves. In stark contrast, *Lotus* species show highly variable PA accumulation in leaves.

The genus *Lotus* includes important forage legumes such as *Lotus corniculatus* L. and *L. tenuis* Waldst et Kit, which belong to a large species complex, called the *L. corniculatus* group. *L. corniculatus* is the most widely grown *Lotus* species worldwide and accumulates PAs [11]. Although sometimes defined to have diploid populations, this species essentially appears to be tetraploid. Biochemical and genetic evidence indicates this species likely arose as a hybrid between *L. uliginosus* and *L. tenuis*[12]. Conversely, *L. tenuis* is diploid and accumulates barely detectable levels of PAs in leaves [13]. Nevertheless, *L. tenuis* is regarded as a “keystone species” for cattle nutrition in areas such the Argentinean Pampas in South America [11], frequently subjected to flooding [14]. In fact, varieties of *L. tenuis* are more tolerant to waterlogging, alkaline and salt conditions than any commercial varieties of *L. corniculatus*.

**Figure 1:**
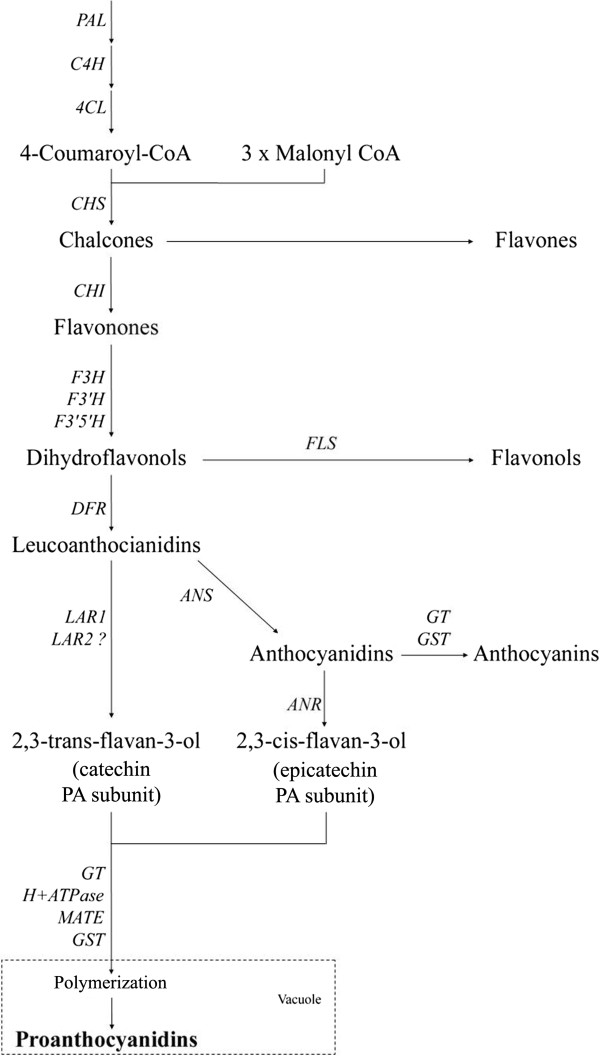
**The flavonoid pathway leading to proanthocyanidins (PAs).** Italic represent the following enzymes: *PAL*: L-Phenylalanine ammonialyase; *C4H*: cinnamate 4-hydroxylase; *4CL*: 4-coumarate:CoA ligase; *CHS*: chalcone synthase; *CHI*: chalcone isomerase; *F3H*: flavanone 3-hydroxylase; *F3*’*H*: flavonoid 3’hydroxylase; *F3*’*5*’*H*: flavonoid 3’5’ hydroxylase; *FLS*: flavonol synthase; *DFR*: dihydroflavonol reductase; *LAR*: leucoanthocyanidin reductase; *ANS*: anthocyanidin synthase; *ANR*: anthocyanidin reductase; *GT*: glucosyltransferase; *GST*: glutathione-*S*-transferase; MATE: multidrug and toxic compound extrusion transporter. A question mark indicates the putative activity of the protein coded by *LAR2*.

Proanthocyanidins are oligomeric and polymeric end-products of the flavonoid biosynthetic pathway synthesized through the catechin and epicatechin branches in most crop species (Figure 1). Most structural genes of the PA pathways have been cloned and characterized in different model crop species, legumes included [15]. The MYB–bHLH–WD40 (MBW) complex regulates the expression of flavonoid biosynthesis genes at the transcriptional level in *Arabidopsis thaliana*[16] and seems to be involved in flavonoid biosynthesis in all plant species. Three regulators, namely TT2 (R2R3MYB), TT8 (bHLH) and TTG1 (WD40 protein), play a central role in the transcriptional regulation of PA biosynthesis in *A. thaliana*. Within this complex, TT2 is the key component regulating PA biosynthesis [17,18] and orthologs of this gene have recently been cloned from *Lotus japonicus*, *Medicago* and *Trifolium spp*[19-21]. Despite this wealth of knowledge, attempts to increase leaf PA content in *Medicago*, *Trifolium* and *Lotus* genotypes to levels sufficient to prevent ruminal bloating by ectopic expression of PA structural and regulatory genes, have all proved unsuccessful [10,21]. Conversely, either ectopic expression of *MYB* repressor (*FaMYB1*) or transgene–mediated gene silencing triggered by a maize *bHLH* gene (*Sn*) were sufficient to deplete, or even switch off the PA pathway in *Lotus* genotypes containing high PA levels in their mesophyll [22-24].

Overall, the lack of variability in leaf PA traits within the most important species, coupled with difficulties in engineering this pathway by transgenesis, prompted us to pursue alternative strategies to enhance this trait in forage legumes of agronomic interest. Introgression of traits between species has received a widespread attention, since interspecific hybridization can lead to the generation of novel molecular and morphological phenotypes that cannot be produced by conventional intraspecific crossing [25,26]. Thus, we exploited interspecific hybridization as a means of creating new gene assortments within the genus *Lotus* to produce genotypes with adequate PA levels in edible tissues. We sought to do so without affecting positive parental traits, such as forage yield and tolerance to environmental stresses. Thus far, the production of *L. tenuis* x *L. corniculatus* hybrids has been hampered by the difference in the ploidy between these species [27].

To overcome this obstacle, we crossed *L. tenuis* plants, from a population selected to grow in marginal areas of South America, with a wild, diploid population of *L. corniculatus* that accumulates PAs in leaves, and which grows in an alkaline-salty area in Spain. The recovery of wild *L. corniculatus* germplasm and its use in an interspecific cross have allowed us to produce *Lotus* hybrids with suitable PA levels in edible organs which are of potential agronomic use. The study of these hybrids and their progeny provides insights into the genetics of PA biosynthesis in legumes.

## Results

### Morphological and molecular characterization of a wild diploid population of *L. corniculatus*

**Figure 2:**
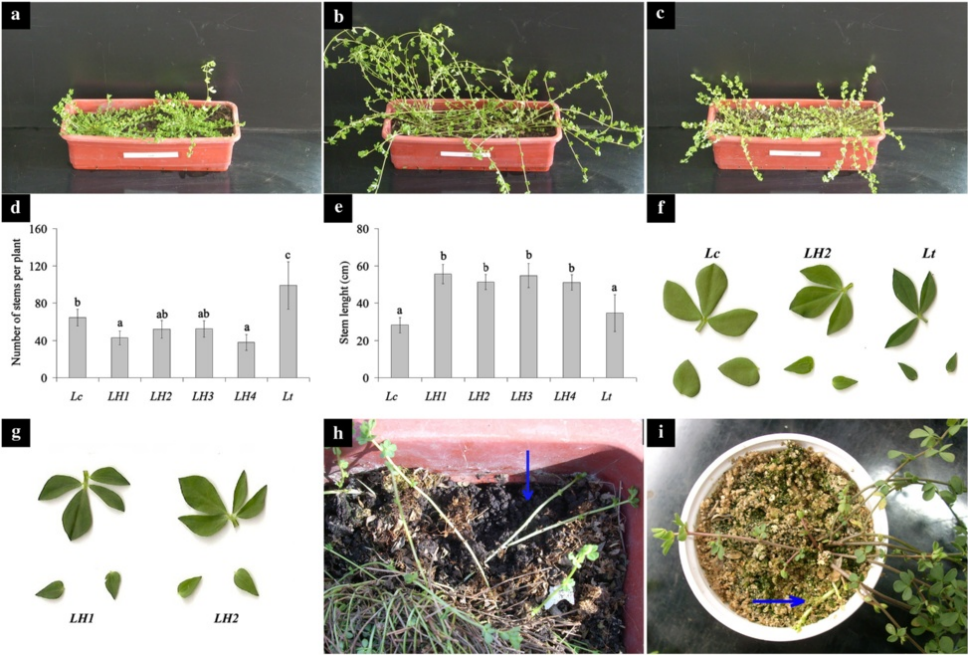
**Morphological characteristics of *Lotus *spp. (a to c) plants grown for a month under glass-house. (a)***L. corniculatus*. **(b)***Lotus tenuis* x *Lotus corniculatus* hybrid. **(c)***L. tenuis*. **(d)** number of stems per plant. **(e)** stem length, *Lc*: *L. corniculatus*; *LH1*, *LH2*, *LH3* and *LH4*: *L. tenuis* x *L. corniculatus* hybrid plants 1, 2, 3, and 4, *Lt*: *L. tenuis*, bars indicate standard deviation of mean values, means with similar letter do not differ significantly (p < 0.05). **(f)** leaves of *Lc*: *L. corniculatus*, *LH2*: hybrid plant and *Lt*: *L. tenuis*. **(g)** leaves of hybrid plants 1 and 2 (*LH1* and *LH2*). **(h and i)** rhizome formation in one-year-old plants, the blue arrow indicates the rhizome. **(h)***L. corniculatus* plant. **(i)** hybrid plant (*LH2*).

*Lotus* plants of the wild population from the Devesa del El Saler in Valencia (Spain) were previously classified as *L. corniculatus*[28]. Morphological traits of the vegetative and reproductive structures of the plants from this population were in fact, with a few exceptions, the same as those of the *L. corniculatus* subsp. *corniculatus* species described by Valdés [29] (Additional file 1: Table S1). However, the wild Spanish population differed from the *L. corniculatus* subsp. *corniculatus* for a number of traits, such as rhizome and stolon production capacity (Figure 2), higher leaf PA content (see below) and diploidy (2n = 12) (Additional file 2: Figure S1). Indeed, all these traits are exhibited by *L. uliginosus*; however plants of this species display fistulous stems, whereas the wild *L. corniculatus* stems were solid. Additional file 1: Table S2 reports the main morphological differences among *Lotus* species, including the wild Spanish population.

For molecular confirmation of the identity of the *Lotus* ecotype found in Spain, genomic DNA was isolated from a number of plants and PCR amplified using the ribosomal primers ITS1/ITS4. Direct sequencing analysis of the ITS1/ITS4 amplicons from all these samples [GenBank: KF164611] gave rise to a 612 bp-long fragment (s) with most samples showing three SNPs (single nucleotide polymorphism) at position 82 (Y), 417 (S) and 505 (M). Similarity search analysis showed 99% identity with the ITS sequence of *L. alpinus* and 96% with that of tetraploid *L. corniculatus*. A dendrogram was built using the ITS sequences of several *Lotus* species retrieved from public databases including the ITS sequence of *L. tenuis* used in this work [GenBank: KF164612]. As shown in Additional file 2: Figure S2, the sequences of the diploid *L. corniculatus* clustered within the *L. corniculatus* group. According to Degtjareva *et al*. [30] not only *L. alpinus* but also *L. tenuis*, *L. corniculatus* and *L. japonicus*, among others, cluster in this group, separated from those of other diploid and PA-rich *Lotus* species (namely *L. uliginosus* also known as *L. pedunculatus*). Thus, molecular data corroborated the morphologically-based classification of the wild diploid *Lotus* as a population belonging to the *L. corniculatus* group. However, they did not provide a definitive evidence on its species identity. Nevertheless, based on morphological analyses carried out here and in a previous study [28], we assigned this population to the *L .corniculatus* species. The seeds from this population were deposited at the Germplam Bank of the Botanical Garden in Valencia under the following code ES-O-VAL-158B2009. For the sake of brevity, we will hereafter refer to the *L. corniculatus* ES-O-VAL-158B2009 population as diploid *L. corniculatus*.

### Production and characterization of *Lotus* interspecific hybrids

Manual crosses between the diploid *L. corniculatus* and *L. tenuis* in both directions were highly efficient and more than 70% of fertilized flowers formed pods. The number of seeds per pod ranged from five to nine, as in parental plants. Seeds obtained by crossing were normal in size and appearance. They were vigorous, with a germination rate higher than 90% in a week.

Resulting seedlings were transplanted into the pots, where they showed a growth rate similar to *L. tenuis* and *L. corniculatus* plants. We obtained more than 50 *L. tenuis* x *L. corniculatus* hybrid plants. All these plants were evaluated for PA accumulation in leaves and stems and all hybrid plants showed an intermediate level of PAs with respect to the parental ones (see below). For further analyses, four representative hybrids, all from the same cross, were randomly selected.

Karyological analysis of the selected hybrids proved that all were diploid (2n = 12) (Additional file 2: Figure S1). Furthermore, ITS sequence analysis was carried out to check the arrangement of this phylogenetically informative marker in the hybrids. Some diagnostic deletions and SNPs were observed between the *L. tenuis* and *L. corniculatus* ITS regions (Additional file 2: Figure S3). In contrast to the parents, the direct sequencing of ITS amplicons from the hybrids produced ambiguous electropherograms. For this reason, they were cloned and individual clones per plant randomly selected and sequenced. Each *L. tenuis* x *L. corniculatus* genotype displayed two ITS patterns, one specific to the *L. tenuis* parent, the other to *L. corniculatus*. These analyses proved the hybrid status of the F1 plants (Additional file 2: Figure S3).

### *Lotus* interspecific hybrids showed agronomically relevant traits

#### a) Morphological evaluation and assessment of forage productivity

Hybrid plants were larger in size and more erect and bulky than either parent (Figure 2a, b and c). Hybrids exhibited a reduction in the number of stems per plant with respect to *L. tenuis*, but an increase in stem length if compared to both parents (Figure 2d and e).

No differences in the area of the trifoliate leaflets were observed between parental plants, or between hybrids. However, the total leaf area of hybrid plants was intermediate in 3 out of the 4 hybrids with respect to *L. tenuis* (1.50 cm^2^) and *L. corniculatus* (1.83 cm^2^). The two parents differed from each other in basal leaflet area (Figure 2f and Additional file 1: Table S3). The length/width ratio of the central leaflets also differed between parental plants; *L. corniculatus* showed wider leaflets with respect to *L. tenuis*, and this trait was intermediate in hybrids (Additional file 1: Table S3). Hybrid plants quite commonly exhibited an additional leaflet, generally found just below the trifoliate leaves (Figure 2g).

The number of leaf trichomes in the *L. corniculatus* genotype was 33/cm^2^, lower than that observed for *L. tenuis* plants (208/cm^2^), while in the F1 hybrids their number ranged from 64 to 112/cm^2^.

During the four harvests, in spring and summer, the total epigeous biomass (shoots) and leaf/stem dry weight ratio in three of the four hybrids analyzed did not differ significantly from the *L. tenuis* parent; only LH4 showed a biomass production similar to *L. corniculatus* but also the lowest leaf/stem dry weight ratio. Biomass production was differently partitioned between the parents: in *L. corniculatus* the contribution of leaves was twice that of stems, whereas in both *L. tenuis* and the hybrids, stems contributed almost as much as leaves to the total biomass (Additional file 1: Table S4).

Moreover, *L. corniculatus* parental plants also displayed the capacity to form rhizomes when grown in pots (Figure 2h). This agronomically relevant trait, which was absent in *L. tenuis*, was inherited by all hybrid plants (Figure 2i).

#### b) Chlorophyll, anthocyanin and PA accumulation

As the leaves of *L. corniculatus* were paler in color than those of *L. tenuis*, the chlorophyll content was measured in both parents and their hybrids. Leaf chlorophyll content was intermediate in hybrids compared to both parents, but chlorophyll content in LH2 was similar to that in *L. tenuis*, the parent with the highest value (Additional file 2: Figure S4).

Concerning anthocyanins, a few anthocyanin-accumulating cells were detected at the base of the trifoliate leaves in both the diploid *L. corniculatus* and hybrid plants, whereas none or very few of these cells were present in *L. tenuis* (data not shown). As *Lotus* spp. primarily accumulate anthocyanins in stems, they were quantified in these organs. As shown in Additional file 2: Figure S4b, anthocyanin content in the hybrids was in between that of the two parental lines, with *L. corniculatus* showing the highest content.

**Figure 3:**
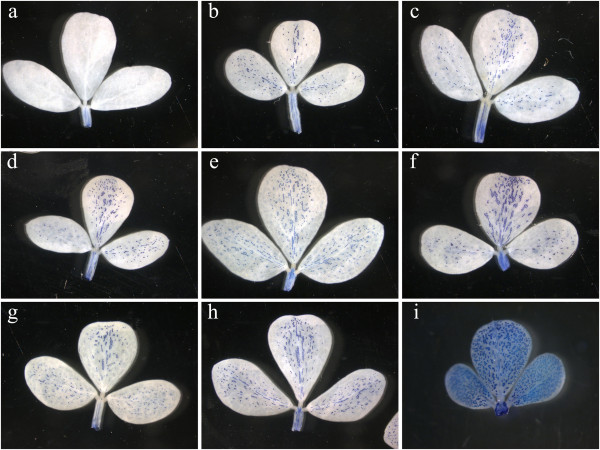
**Patterns of PA accumulation in *Lotus *spp.** Leaves from **(a)***L. tenuis*, **(b to h)** hybrid plants and **(i)***L. corniculatus* stained with DMACA.

To compare the distribution patterns and content of PAs in parental and hybrid genotypes, histological and spectrophotometric analyses were carried out. PA-accumulating cells in *Lotus tenuis* were present only in the vascular tissues of leaves and petioles (Figure 3a). In contrast, *L. corniculatus* presented PA-accumulating cells throughout the leaf blade, as well as in the vascular tissue (Figure 3i). Hybrid plants showed a similar PA pattern accumulation in the leaves as compared to *L. corniculatus*, although the number of PA-accumulating cells in the leaf blade was markedly lower with respect to the paternal partner (Figure 3b to h). Results of PA quantification from parents and hybrids are given in Table 1. The low number of PA-accumulating cells in *L. tenuis* leaves resulted in a lower PA levels (less than 1 mg PA/g DW) compared with levels in both *L. corniculatus*, which accumulated about 25 mg PA/g DW, and the hybrids. The average value of leaf PAs in the 50 hybrid plants was 5.2 ± 0.9 mg PA/g DW. Thus, leaf PA content in hybrids was intermediate between the parents. A similar result was obtained when the F1 population from the reciprocal cross (*L. corniculatus* x *L. tenuis*) was assayed (4.7 ± 1.3 mg PA/g DW). In stems, PA levels were lower than in leaves for all evaluated genotypes (p < 0.01). However, PA levels in stems paralleled those of leaves in that PA content in the hybrids was intermediate between parents with *L. corniculatus* accumulating over six times more total PAs than *L. tenuis* (Table 1). A similar trend was exhibited in flowers, which were the organs that displayed the highest levels of PAs among all the genotypes (Table 1).

**Table 1:** PA content in leaves, stems, flowers and roots of parental and F1 hybrid plants

**Sample**		**Soluble proanthocyanidins**					**Insoluble proanthocyanidins**					**Total proanthocyanidins**				
		**Mean**		**S.D.**	**Ranks**		**Mean**		**S.D.**	**Ranks**		**Mean**		**S.D.**	**Ranks**	
**Leaves**	***Lc***	10.84	±	1.51	65.5	**e**	12.3	±	4.14	63.5	**c**	25.85	±	4.85	65.5	**d**
	***LH1***	1.08	±	0.18	41.6	**cd**	4.71	±	0.98	37.3	**b**	5.79	±	0.94	37.8	**bc**
	***LH2***	0.58	±	0.30	25.7	**bc**	4.25	±	1.58	32.3	**b**	4.83	±	1.67	29.8	**b**
	***LH3***	0.50	±	0.12	24.0	**b**	3.97	±	1.92	29.3	**b**	4.47	±	1.91	27.8	**b**
	***LH4***	1.71	±	0.58	50.8	**de**	5.86	±	1.99	45.2	**b**	7.58	±	2.42	46.7	**c**
	***Lt***	0.06	±	0.02	6.0	**a**	0.93	±	0.34	6.0	**a**	0.99	±	0.34	6.0	**a**
**Stems**	***Lc***	0.37	±	0.16	52.5	**d**	2.49	±	1.15	46.9	**c**	2.82	±	1.13	48.5	**c**
	***LH1***	0.07	±	0.04	23.7	**abc**	1.16	±	0.70	24.6	**b**	1.23	±	0.73	24.3	**b**
	***LH2***	0.11	±	0.06	31.2	**bc**	1.47	±	0.41	36.5	**bc**	1.58	±	0.42	36.0	**bc**
	***LH3***	0.14	±	0.06	36.8	**c**	1.07	±	0.35	24.9	**b**	1.20	±	0.36	26.3	**b**
	***LH4***	0.05	±	0.04	17.1	**ab**	1.07	±	0.24	26.7	**b**	1.11	±	0.26	24.6	**b**
	***Lt***	0.03	±	0.01	11.1	**a**	0.40	±	0.04	6.5	**a**	0.43	±	0.04	6.3	**a**
**Flowers**	***Lt***	12.43	±	2.36		**d**	14.55	±	2.79		**c**	26.97	±	0.76		**c**
	***LH1***	9.14	±	1.84		**bc**	11.97	±	4.07		**bc**	21.11	±	5.87		**bc**
	***LH2***	9.62	±	0.14		**cd**	14.17	±	2.61		**c**	23.80	±	2.59		**c**
	***LH3***	6.19	±	1.57		**ab**	7.65	±	4.11		**ab**	13.84	±	2.94		**a**
	***LH4***	8.97	±	1.47		**bc**	6.78	±	1.96		**ab**	15.74	±	3.21		**ab**
	***Lc***	3.88	±	2.22		**a**	5.70	±	3.18		**a**	9.58	±	4.33		**a**
**Roots**	***Lc***	0.46	±	0.13		**a**	0.65	±	0.10		**b**	1.12	±	0.19		**ab**
	***LH1***	0.82	±	0.34		**bc**	0.72	±	0.11		**bc**	1.54	±	0.38		**bc**
	***LH2***	0.86	±	0.18		**c**	0.66	±	0.17		**bc**	1.52	±	0.34		**bc**
	***LH3***	0.47	±	0.18		**a**	0.36	±	0.14		**a**	0.83	±	0.30		**a**
	***LH4***	0.50	±	0.10		**ab**	0.36	±	0.14		**a**	0.86	±	0.21		**a**
	***Lt***	0.74	±	0.18		**bc**	0.96	±	0.10		**c**	1.70	±	0.26		**c**

Samples are as in Figure 2. Values are expressed as mg PA g-1 of dry weight ± standard deviation. Within each organs block means with a similar letter do not differ significantly (p < 0.05).

Root PA levels showed a contrasting profile with respect to those of epigeous organs: *L. tenuis* accumulated significantly higher amounts of these compounds than *L. corniculatus*. LH3 and LH4 hybrids displayed a lower level of root PAs than LH1 and LH2. Nevertheless, all hybrids did not differ from *L. corniculatus* for this trait (Table 1).

TLC analysis showed that the anthocyanidins released by buthanol:HCl hydrolysis of PAs extracted from the leaves of the hybrids were cyanidins and delphinidins. A similar pattern was exhibited by the parental *L. corniculatus* genotype, whereas the levels of PAs from *L. tenuis* leaves were under the TLC detection limit (Additional file 2: Figure S5).

#### c) Nutritional value of the hybrids

Total proteins in LH1, LH2, LH3 and LH4 were 21.4, 22.1, 22.3 and 21.6% of the dry matter, respectively. In *L. tenuis* and *L. corniculatus* they were 23.2% and 16.2%, respectively. The *in vitro* dry matter digestibility (IVDMD) in the hybrids was similar to or even higher (81.0; 84.3; 81.0 and 80.4% in LH1, LH2, LH3 and LH4, respectively) than in *L. tenuis* (80.2%), whereas it was lower in *L. corniculatus* (78.1%).

### *Lotus* hybrid plants as a model to study genetic regulation of PA biosynthesis

#### a) Cloning and expression analysis of PA biosynthetic and regulatory genes

Genomic and/or cDNA fragments relative to structural (*PAL*, *CHS*, *DFR*; *ANS*, *ANR*, *LAR1* and *LAR2*) and regulatory (*R2R3MYB* and *bHLH*) genes of the PA pathway were amplified and sequenced from both parents. Likewise, the *EF*-*1α* gene used as housekeeping gene in qRT-PCR analysis. All the amplified genes showed high sequence identity (>93%) to the predictive orthologs and between the two parents (Additional file 1: Table S5). Because most of these genes are arranged as small multigene families in *Lotus* spp. [19,23,31,32] for each target gene the primers for qRT-PCR analysis were designed on highly conserved regions within and between the *L. tenuis* and the diploid *L. corniculatus* genotypes.

**Figure 4:**
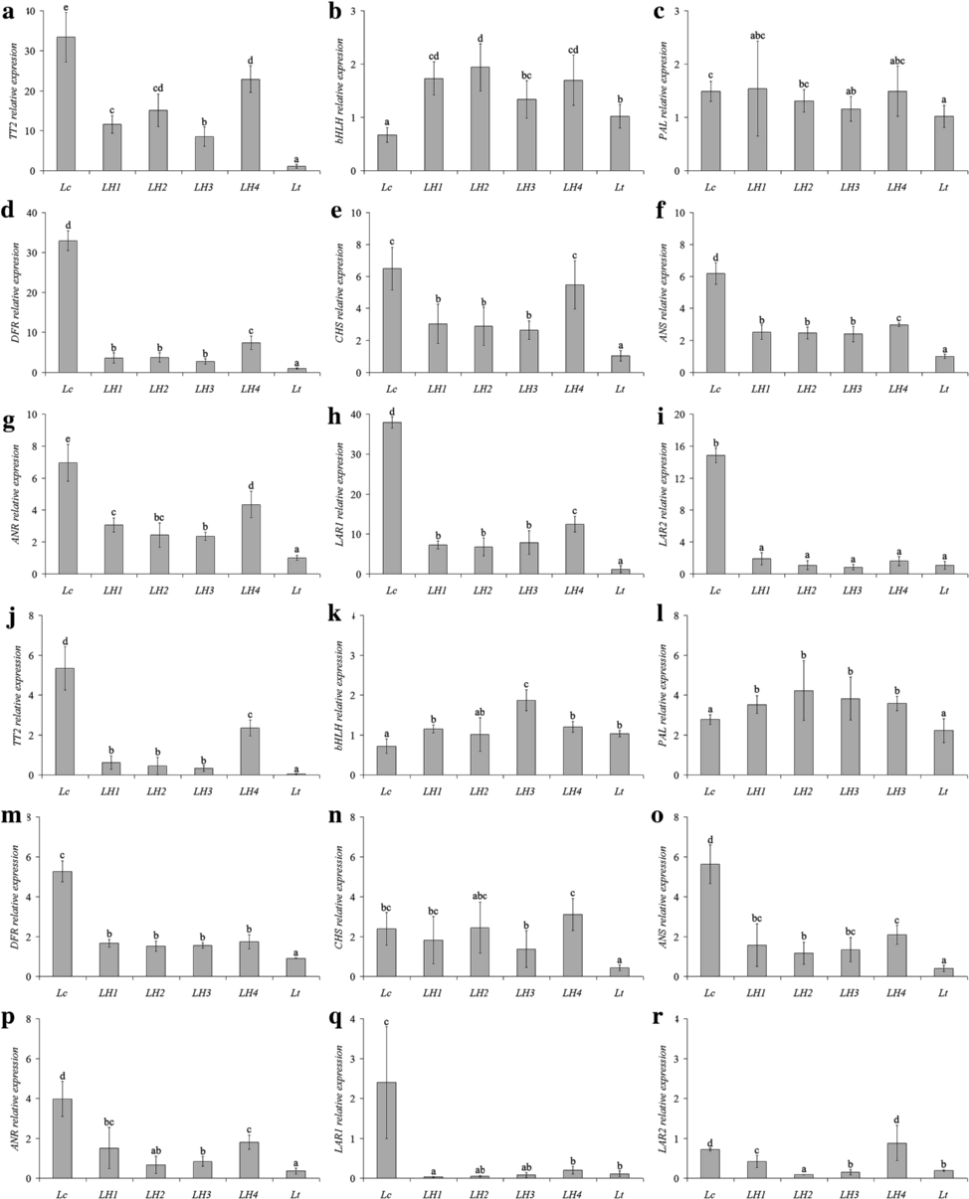
**Relative expression of PA genes in parental and F1 hybrid plants. (a to i)** leaves. **(j to r)** stems. Expression was determined by real-time RT-PCR analysis using *EF*-*1α* as the housekeeping gene. Ct value of *L. tenuis* leaves was arbitrarily selected as reference. Samples are as in Figure 2. Means with similar letter do not differ significantly (p < 0.05), bars indicate standard deviation of mean values.

In leaves, of the two early PA biosynthesis genes tested by qRT-PCR, namely *PAL* and *CHS*, only the latter one displayed a marked difference between parents. *CHS* expression was also intermediate in the hybrids (Figure 4). When the late PA biosynthesis genes were assayed, it turned out that all the genes, except *LAR2*, displayed an expression profile that resembled that of *CHS* in that their expression levels were the highest in *L. corniculatus*, the lowest in *L. tenuis* and intermediate in the hybrids. Among these latter genotypes LH4 displayed the highest expression levels for all the genes tested. Conversely hybrids did not differ from *L. tenuis* for the steady state levels of *LAR2* (Figure 4).

Among the ternary transcriptional MBW complex that controls flavonoid biosynthesis in plants, the *R2R3MYB* and *bHLH* partners appeared to be rate-limiting factors for PA accumulation in *Lotus* spp. [31]. Orthologs of *TT2*, the *R2R3MYB* gene that specifies PA accumulation in *Arabidospis thaliana* seeds, cloned from a tetraploid *L. corniculatus* plant [23] and the model species *L. japonicus*[32] provided sequence information to successfully amplify a partial transcript of this gene from RNA isolated from leaves of the diploid *L. corniculatus* parent (Additional file 1: Table S5). Conversely, no amplification was obtained for leaf or stem cDNA from the *L. tenuis* plants. However, when the same primers were used to amplify *L. tenuis* genomic DNA a *TT2*-specific fragment was obtained (data not shown). Overall, the data suggested that the *TT2* gene was barely expressed or not expressed at all in *L. tenuis* leaves and stems. In keeping with this observation, qRT-PCR analysis proved that *TT2* was highly expressed in the PA-rich diploid *L. corniculatus* parent but far less (over 30 times less) in *L. tenuis*. All four hybrids exhibited a *TT2* steady state level higher than *L. tenuis*, from about 10 (LH3) to more than 20-fold (LH4). Notably, the dissociation analysis followed by sequencing of the qRT-PCR products obtained using *TT2*-specific primer pairs proved the co-amplification of at least two *TT2* gene members in *L. corniculatus* and in the hybrids (data not shown).

When the present study began, *TAN1* cloned from *L. uliginosus* and *L. japonicus* was the only *Lotus* spp. sequence relative to the *bHLH* gene, putatively associated with PA biosynthesis, present in public databases. An ortholog of this gene was amplified and sequenced from the leaf cDNA of both parents. Sequencing analysis did not reveal the amplification of different gene members within each parent and showed a high similarity between these cDNAs and that of *L. japonicus* (99% sequence identity) (Additional file 1: Table S5). Primers were then designed to test the expression of this *bHLH* gene in both parental and hybrid plants. The steady state levels of *TAN1* gene were higher in *L. tenuis* and in the four hybrids plants than in the diploid *L. corniculatus* (Figure 4).

The expression patterns of all these genes in the stems of the genotypes under investigation were similar to those observed in leaves, although with few exceptions (Figure 4). Indeed, the expression levels of *LAR1* in the stems of the hybrids were similar or even lower (LH1) than in stems of *L. tenuis*, whereas *LAR2* did not show a clear expression pattern in the hybrids (Figure 4).

Statistical analyses proved a positive correlation between the expression of *CHS*, *DFR*, *ANS*, *ANR*, *LAR1* and *LAR2* genes, with the overall expression of the *TT2* transcriptional factor in the leaves and stems of the different genotypes evaluated. Notably, the expression of *TT2* highly correlated with accumulation of PAs in leaves and stems of parental and hybrid plants (Table 2). In stark contrast, expression of the *bHLH* gene *TAN1* did not correlate with PA levels in either stems or leaves, nor with expression levels of the structural genes (Table 2).

**Table 2:** **Correlation analyses between the relative expression** (**2**^-**ΔΔCt**^) **of the biosynthetic and regulatory PA genes and the PA content** (**mg PAs g**^-**1 **^**of dry weight**); **Pearson test**

**Leaves and stems of parental and F1 hybrids**										
**Gene**	**Soluble PAs**		**Insoluble PAs**		**Total PAs**		***TT2***		***bHLH***	
	**Correlation coefficient**	**p-value**	**Correlation coefficient**	**p-value**	**Correlation coefficient**	**p-value**	**Correlation coefficient**	**p-value**	**Correlation coefficient**	**p-value**
***TT2***	0.83	0.0008	0.97	<0.0001	0.91	<0.0001				
***bHLH***	-0.36	0.2500	-0.11	0.7500	-0.25	0.4200				
***PAL***	-0.31	0.3300	-0.50	0.1000	-0.40	0.2000	-0.49	0.0010	-0.002	0.90
***CHS***	0.78	0.0029	0.90	<0.0001	0.85	0.0005	0.79	<0.0001	0.07	0.68
***DFR***	0.99	<0.0001	0.92	<0.0001	0.98	<0.0001	0.82	<0.0001	-0.27	0.09
***ANS***	0.70	0.0100	0.76	0.0045	0.74	0.0100	0.70	<0.0001	-0.30	0.05
***ANR***	0.82	0.0010	0.92	<0.0001	0.88	0.0002	0.89	<0.0001	-0.18	0.26
***LAR1***	0.97	<0.0001	0.98	<0.0001	0.99	<0.0001	0.91	<0.0001	-0.23	0.15
***LAR2***	0.99	<0.0001	0.89	<0.0001	0.97	<0.0001	0.78	<0.0001	-0.32	0.04
**Leaves of parental and selected F2 plants**										
**Gene**	**Soluble PAs**		**Insoluble PAs**		**Total PAs**		***TT2***		***bHLH***	
	**Correlation coefficient**	**p-value**	**Correlation coefficient**	**p-value**	**Correlation coefficient**	**p-value**	**Correlation coefficient**	**p-value**	**Correlation coefficient**	**p-value**
***TT2***	0.85	<0.0001	0.86	<0.0001	0.87	<0.0001				
***bHLH***	-0.31	0.1400	-0.15	0.5000	-0.21	0.3200				
***PAL***	-0.29	0.1800	-0.19	0.3800	-0.23	0.2800	-0.01	0.9500	0.29	0.1700
***CHS***	0.41	0.0500	0.41	0.0400	0.42	0.0400	0.29	0.1700	0.08	0.7100
***DFR***	0.87	<0.0001	0.81	<0.0001	0.85	<0.0001	0.71	0.0001	-0.32	0.1300
***ANS***	0.79	<0.0001	0.80	<0.0001	0.81	<0.0001	0.88	<0.0001	-0.28	0.1900
***ANR***	0.71	0.0001	0.75	<0.0001	0.75	<0.0001	0.88	<0.0001	-0.18	0.4100
***LAR1***	0.89	<0.0001	0.91	<0.0001	0.92	<0.0001	0.89	<0.0001	-0.12	0.5800
***LAR2***	0.83	<0.0001	0.84	<0.0001	0.85	<0.0001	0.66	0.0004	-0.47	0.0200

#### b) Production and analysis of F2 populations

An F2 population was obtained to gain greater insight into genetic inheritance of the PA trait. Phenotypically, plants of this population were widely variable: some displayed traits, such as stem number and stem length, similar to *L. tenuis* or *L. corniculatus*, while others were similar to *L. tenuis* x *L. corniculatus* plants (data not shown). Conversely, the capacity to form rhizomes was observed in the majority of plants from F2 populations.

Depending on the number of PA-containing leaf cells, the 200 plants were sorted into 5 groups (Additional file 2: Figure S6). Interestingly, none of the F2 genotypes varied outside the range of PAs of the parental genotypes. The most representative group was Class 1, clustering plants with the lowest number of PA-containing cells; conversely, the least representative group was Class 5, clustering plants with the highest number of PA-containing cells.

Three F2 plants belonging to Class 1 and three to Class 5 were then assessed for PA accumulation in leaves. The average value of leaf PAs in Class 1 plants (3, 5, 147) was 0.81 ± 0.3 mg PAs/g DW, similar to *L. tenuis* (0.65 ± 0.2 mg PAs/g DW), while under the same conditions, the corresponding value for Class 5 plants (27, 120, 186) was 19.88 ± 5.9 mg/g DW, and that of the diploid *L. corniculatus* parent was 30.99 ± 0.6 mg PAs/g DW.

Although Class 5 plants displayed as much pigmentation over whole the leaf blade as *L. corniculatus*, none of these plants showed PA values similar to the paternal genotype, suggesting that the quantitative difference in PA levels does not reside solely in the number of pigment-accumulating cells but also in the levels of these pigments in each cell.

Plants from Class 1 and 5 revealed marked differences in the expression levels of all the genes tested, with the exception of *PAL* and *bHLH*. More specifically, plants in these two groups exhibited expression differences exceeding 2× for *ANS*, *ANR*, *LAR1*, *LAR2* and *TT2* genes (Additional file 1: Table S6). Correlation analyses revealed a strong linear relationship between *TT2* and *ANS*, *ANR* and *LAR1* genes, as well as between all these genes and PAs when data from F2 plants and parental plants were compared (Table 2).

## Discussion

Mitigating the negative environmental impacts of pastoral animal-production systems by improving the nutritional values of forage legumes has become a major goal for breeders worldwide. Accumulation of moderate amounts of PAs in the most valuable forage legumes belonging to the *Medicago*, *Trifolium* and *Lotus* genera can help enhance ruminant productive performance while simultaneously decreasing environmental costs [4,33]. Unfortunately, the lack of genetic variability for this trait and the complexity of its genetic control have so far prevented forage breeders from achieving this goal [34]. Thus, we decided to exploit a wild, diploid *L. corniculatus* population, which grows in a marginal environment and synthesizes PAs in leaves, to cross *L. tenuis* and produce interspecific hybrids with adequate PA levels in edible organs. To the best of our knowledge the *L. tenuis x L. corniculatus* hybrids, obtained in this work, represent the first example of the introgression of the PA trait in forage legumes to levels known to provide nutritional and health benefits to ruminants (around 5 mg PAs/g DM). Not only do these hybrids retain traits of agronomic relevance from both parents, but their analysis also shed light on the inheritance and genetic control of leaf PAs in legumes. Furthermore, the diploid *L. corniculatus* population is instrumental to addressing phylogenetic relationships within this genus and the interspecific hybrids and their progeny pave the way to mapping QTLs related to PAs and tolerance to salt stress.

### The wild, diploid and PA-rich *L. corniculatus* population is of relevance for phylogenetic studies and represents a source of novel genetic traits

The *Lotus* genus is found worldwide and consists of about 100–130 annual or perennial species with a basic chromosome number n = 5, 6, 7. As species complexes with similar taxonomical traits are present, the taxonomy within this genus is far from being clearly resolved [12]. As an example, the origin of tetraploidy (2n = 4 × = 24) in *L. corniculatus* and *L. alpinus* have yet to be clarified [35,36]. The wild and PA-rich *Lotus* population described here is diploid (2n = 2 × = 12) and morphologically resembles the tetraploid *L. corniculatus* for many taxonomical traits of both vegetative and reproductive structures and organs (Figure 2 and Additional file 1: Table S3 and S4). However, unlike this species, the wild diploid *Lotus* exhibits rhizomes and stolons, organs formed by *L. uliginosus*, another PA-rich diploid species [29]. Along with morphological observations, ITS-based screening also clusters this population within the *L. corniculatus* group (Additional file 2: Figure S2). In addition to *L. corniculatus*, this group encompasses as many as 12 diploid and tetraploid species, including *L. alpinus* and *L. tenuis*, but not *L. uliginosus*[30]. The ITS sequence of the diploid *L. corniculatus* is more similar to the ITS sequence from *L. alpinus* (99% of identity) than that of the tetraploid *L. corniculatus* (96%); however, the morphology of *L. alpinus* differs from that of the diploid *L. corniculatus* for many traits (Additional file 1: Table S2). Yet, *L. alpinus* does not contain PAs in the leaves [37] and there are both diploid and tetraploid accessions within this species. It is unknown whether the *L. alpinus* ITS sequence present in GenBank database is from a diploid or tetraploid accession. Notably, previous studies based on polymorphism of the β-*tubulin* gene have shown that tetraploid accessions of *L. alpinus* and *L. corniculatus* share common bands, and that tetraploid *L. alpinus* accessions do not originates from an autotetraploidization event but likely from crosses between two diploid species [12]. Overall, due to the paucity of phylogenetically informative markers from a number of different diploid and tetraploid populations of *L. alpinus* and *L. corniculatus* inferring which is the closest relative to the diploid *Lotus* population described in this study is difficult. However, combining molecular and morphological data sets from these taxa should enable us to resolve this issue. Additionally, the cross of the wild diploid *Lotus* population with ecotypes and commercial varieties of diploid *Lotus* spp. will allow us gleaning more insights into the species boundaries within the *Lotus* genus. While waiting for more definitive genetic evidence, based on present findings and an existing report [28], we argue that the morphological analysis is informative enough to assign the wild diploid Spanish population to the *L. corniculatus* species.

Notably, the diploid *L. corniculatus* population accumulates twice as much PAs as the commercial *L. corniculatus* variety [13]. It grows in a harsh environment like the Devesa de El Saler in Valencia (Spain) where stress-tolerant genotypes of numerous plant species have been recovered [38]. Thus this population is a potential donor parent of various traits to breed improved forage legumes in terms of adaptability to marginal areas.

### F1 hybrids of *L. tenuis* and the wild diploid *L. corniculatus* have a forage yield comparable to the cultivated parent

Artificial hybridization brings together genotypes that would not normally meet in nature as result of genetic, ecological or geographic barriers. Within *Lotus*, interspecific hybridization in the genus *Lotus* has been performed employing tissue culture and molecular techniques at several experimental centers [27]. *L. tenuis* x *L. corniculatus* hybrids have been obtained by crossing tetraploid accessions of both species or male sterile 4 × *L. corniculatus* with a diploid *L. tenuis*, producing unreduced gametes [27]. Previous hybridization experiments have aimed to evaluate relationships between these taxa and improve *L. corniculatus* seedling vigor. However, to date, no successful crosses between diploid accessions of these species have been obtained [39]. By contrast, the crosses between the diploid *L. tenuis* and the wild diploid *L. corniculatus* undertaken in this study were successful in both directions and produced fertile F1 hybrids. The *L. corniculatus* parent employed in the present study likely provides a gene pool that differs from those of the *L. corniculatus* genotypes employed in earlier studies. We note that few Institutions worldwide host the genetic resources necessary for *L. corniculatus* improvement [40].

Regarding morphological traits of the *L. tenuis* x *L. corniculatus* hybrids, many proved to be intermediate to corresponding parental characteristics. There were, however, exceptions to this rule. Regardless of the direction of the cross, all F1 hybrids produced rhizomes and stolons as a dominant inherited trait. Similarly, Samek and Beuselink [41] reported that on crossing tetraploid Moroccan *Lotus* plants possessing rhizomatous growth habits and tetraploid *L. tenuis*, the resulting progeny produced rhizomes. The only trait that outcompetes the range observed in the parental genotypes suggesting hybrid vigor is stem length, which is significantly higher in the *L. tenuis* x *L. corniculatus* hybrid than in their parents. Indeed, stem length and rhizome production are traits of agronomic importance as they ensure persistence and competition in pastures [42]. Furthermore, forage yields tested in pot-grown plants were higher in the hybrids than in the diploid *L. corniculatus* and did not differ significantly to *L. tenuis* yield in three out of the four hybrids tested. Likewise, forage production partitioning in the hybrids resembled that of *L. tenuis*, as stems contributed more than leaves in all these plants. These are additional traits that are not under additive control.

Further experiments should be performed to compare the nutritional values of the hybrids versus their parents; however, preliminary data show they have similar total protein content and *in vitro* dry matter digestibility (IVDMD) to the cultivated parent. Notably, Kelman reported significant negative correlations of PAs with IVDMD and N concentrations in accessions of the PA-rich species *L. corniculatus* and *L. uliginosus*[40]. The latter author emphasized the need to obtain *Lotus* accessions with a PA concentration that would be effective in producing bypass protein while minimizing the negative effects of these pigments on palatability and intake. In this respect, the production of interspecific *L. corniculatus* x *L. uliginosus* hybrids with low PA and high IVDMD by Kelman [40] and the *L. tenuis x L. corniculatus* hybrids described here confirm that interspecific hybridization is a valuable tool to improve agronomic and forage quality traits of *Lotus* species.

Regarding parents plants’ habitat, *L. tenuis* is cultivated in the alkaline and flooding-susceptible soils of the Flooding Pampas (Argentine), whereas diploid *L. corniculatus* grows spontaneously in a highly saline and alkaline area in Spain. These two parental lines have been crossed to obtain genotypes that not only retain *L. tenuis* forage yield and synthesize PAs in edible organs, but also retain tolerance to the aforementioned stress conditions. A long-term goal of the present study is to provide a segregating population for all these traits with a view to addressing their genetic control. Interestingly, when challenged with 150 mM NaCl for up to 45 days, *L. corniculatus* and hybrid plants showed far less marked signs of senescence than *L. tenuis* (Additional file 2: Figure S7). These preliminary observations were corroborated by the finding that both *L. corniculatus* and hybrid plants showed a significantly lower chloride content in older leaves than *L. tenuis* (data no shown). In summary, crossing *L. tenuis* and the diploid *L. corniculatus* gives rise to hybrid plants that perform as well as the cultivated parental line for many traits of agronomic relevance, such as forage yield and adaptability to challenging soil conditions.

### *L. tenuis* x *L. corniculatus* F1 hybrids synthesize PAs in foliage and this trait segregates in the progeny

The inheritance of PAs has been studied mainly with reference to their synthesis and accumulation in the seed coat. In the common bean (*Phaseolus vulgaris*), this inheritance fits an oligogenic model [43] and there is evidence for dominance of low PA content in populations derived from crosses between parents with high and low PA content [44]. Less is known about the inheritance of PAs biosynthesis in vegetative organs of legumes. Ross & Jones [45] crossed tetraploid *L. corniculatus* plants with high and low PA levels and visually scored these metabolites by the vanillin-HCL in leaves of F1 plants as well as in the leaves of both F1 plants and the backcross of F1 with the low PA parent. They concluded that foliar PA production was controlled by a monogenic dominant trait, which segregated with tetrasomic inheritance. A similar conclusion was drawn by Darlympe *et al*. [46]; who scored the PA content in F1, F2 and BC populations, derived from the cross of high and low PA-containing parents from two *L. corniculatus* cultivars. They reported that PA inheritance in this plant species is controlled by a single dominant gene, inherited disomically. Conversely, on performing diallel crosses between *L. corniculatus* plants with high, medium and low PA levels, Miller & Ehke [47] observed that PA concentration is controlled primarily by additive genetic effects and proposed the role of major genes with complementary gene action to explain the non additive genetic effects in one diallel set. Here we show that, irrespective of the direction of the cross, the hybrids produced by crossing *L. tenuis* x *L. corniculatus* accumulate PAs, in both leaves and stems, to levels which are intermediate between the parents. No segregation for this trait was observed in F1 but only in F2. We observed that in the F2 population, the most representative class groups plants with PA levels similar to the *L. tenuis* parents (Additional file 2: Figure S6), whereas none of the F2 plants showed PA levels comparable to *L. corniculatus*. The average value of leaf PAs in Class 5 plants, which groups all the progeny with the highest PA score, is around three times lower than *L. corniculatus*. Overall, the data show different PA distribution in the organs of the same species along with a fine tuning of their levels, suggesting that this trait is controlled by a few or even multiple genes with a complex interplay between regulatory genes. To gain greater insight into the main genes related to PA accumulation, we tested for the expression of orthologs of regulatory and biosynthetic flavonoid pathway genes previously cloned from different *Lotus* species in parental lines, F1 and F2 populations. The *bHLH* gene (*TAN1*) apparently involved in this trait, neither correlates with the expression of these genes nor with PA levels. Conversely, a highly significant correlation was observed between *TT2* expression and PAs (Table 2). *TT2* expression also correlates with the expression of genes for PA biosynthesis *ANS*, *ANR*, *LAR1* and *LAR2*. Thus, *TT2* is a major gene for the trait and, in fact, a change in its expression level of just a single relative unit explains 0.5 and 0.4 mg PAs/g DW in F1 and F2 plants. Yoshida *et al*. [19,32] have recently cloned three *TT2* gene members in *L. japonicus* and observed that the master regulator of PA biosynthesis (*LjTT2a*) is only slightly expressed in leaves and stems. The low expression level of this regulator is consistent with the limited capacity of this species to accumulate PAs in edible tissues. Experiments are ongoing to test whether or not the different *TT2* forms expressed in the plants analyzed in the present study, contribute differently to this trait. Nevertheless, present data suggest that *TT2* expression *in toto* is significantly downregulated in *L. tenuis*, likely due to the presence of negative regulators that specifically act on the PA pathway in stems and leaves but not in roots. Consistent with this speculation, is the finding that PA levels increase twofold (10.62 ± 1 vs 5.2 ± 0.68 mg PAs/g DW) when F1 plants are backcrossed with 2× *L. corniculatus* (unpublished results). Indeed, the presence of PA repressors limiting the expression or the activity of PA activators may explain the unsuccessful *de novo* induction of PA biosynthesis in legumes by the ectopic expression of MYB and or bHLH activators [23]. In soybean, the absence of seed pigmentation is controlled by a dominant allele in the _*I*_ locus while the homozygous recessive ii produces a totally pigmented seed coat, the alternate ii allele confines pigmentation to the hilum [48]. Furthermore, Gruber and colleague [49] obtained transposon-tagged *L. japonicus* lines exhibiting PAs and *LAR* activity in their leaves, and suggested a repressor gene might be responsible for blocking flavonoid biosynthesis in legumes such as *L. japonicus*, alfalfa and white clover. Besides the R3 and R2R3MYB repressors of the flavonoid pathway, such as *AtMYBL2* and *FaMYB1* reported to repress PAs in *Arabidopsis* and *L. corniculatus*, respectively [23,50,51], members of the *LBD* (lateral organ boundary domain) gene family negatively affect PA and anthocyanin accumulation by repressing transcription of the *MYB* genes *PAP1* and *PAP2*, at least in *Arabidopsis*[52].

Regarding structural genes of the flavonoid pathway, of the two early genes tested, *PAL* does not correlated with total PAs whereas the expression of *CHS* correlates with PAs and *TT2* in F1 but not in F2 (Table 2). More in depth analyses are underway to dissect the genetic and environmental regulation of the different gene members within these two gene families and assess the contribution of the different isoforms from the two parental genomes to the biosynthesis of PAs and other branch products of the flavonoid pathway. However, on the basis of present results we can infer that the products of these two gene families are not the rate limiting factors for the activity of the downstream enzymes coded by the PA-specific genes *ANR*, *LAR1* and *LAR2*. With respect to these genes, our data reinforce previous hypotheses, based on the ectopic expression of *MYB* or *bHLH* genes, that the parallel catechin and epicatechin PA pathways are tightly co-regulated in *Lotus* leaves [23,31]. Additionally, they hint at the role of *LAR2* in PA biosynthesis. Although *LAR2* has all the domains that characterize leucoanthocyanidin reductases (LAR), no functional evidence for this gene has yet been provided [31]. Unlike other PA structural genes, this gene is unaffected in the tetraploid *L. corniculatus* plants that ectopically express the flavonoid R2R3MYB repressor gene *FaMYB1* from strawberry, and in those that ectopically express the anthocyanin and PA activator *bHLH* gene *Sn* from maize [23,31]. However, in tetraploid *L. corniculatus* transgenic lines, in which *Sn* is silenced and PA synthesis severely decreased, *LAR2* steady state levels are also significantly reduced [31]. Few authors have questioned the involvement of this gene in PA biosynthesis [10]. Interestingly, our study shows that *LAR2* expression correlates with PA accumulation, the expression of *TT2* and that of the other late structural genes tested (*DFR*, *ANS*, *ANR* and *LAR1*). Thus, *LAR2* appears to be intimately related to the biosynthesis of PAs and its expression is likely necessary although insufficient to ensure adequate PA levels in edible organs of *Lotus* spp. It is also conceivable that the *LAR2* regulatory mechanism partially overlaps with those of the *ANR* and *LAR1* branches of the pathway. In summary, the interspecific hybrids and the resulting F2 population segregating for the PA trait have provided us with tools alternative to the production of mutants *via* the ectopic expression of regulatory and/or structural genes to gain insights into the regulation of these pigments in the foliage of forage legumes. This marks a clear-cut difference with respect to previous works, both in terms of the results achieved and the methodology employed. Consequently, to gain a better understanding of PA regulation, *de novo* identification of regulatory genes by QTL and eQTL analyses is currently underway.

## Conclusions

The diploid *L. corniculatus* is a valuable donor parent in forage breeding programs as it has traits of interest, such as the ability to synthesize PAs in herbage, form rhizomes and tolerate salty conditions. In this context, here we show that the interspecific cross between diploid *L. tenuis* and *L. corniculatus* produces an F1 hybrid population whose agronomical value is similar to the cultivated *L. tenuis* parent. Better still, the hybrids outweigh the *L. tenuis* parental line because they accumulate PAs in edible organs to levels known to provide health and nutritional benefits to ruminants and produce rhizomes. Thus, the hybrids and their progeny represent a novel genetic pool to breed superior legume varieties and an invaluable new means to advance our grasping of the genetic control of traits such as PA biosynthesis and salt tolerance. Finally, from a pragmatic point of view as the hybrids between these two species are relatively simple to obtain and propagate through rhizomes, they might be also introduced into cultivated pastures to increase both productivity and nutritional value.

## Methods

### Plant material

*Lotus tenuis* plants used in this study were from a commercial variety, tolerant to Argentinean saline-alkaline soils. *L. corniculatus* plants were from a wild population, recovered from area known as Devesa de El Saler (Latitude 39°20′41″ N; Longitude 00°19′12″ W, Valencia, Spain). Taxonomical determination of the recovered wild *L. corniculatus* population was carried out according to morphological traits of vegetative and reproductive organs, as described in Valdés [29].

Seeds of both populations were scarified and sown according to Escaray *et al*. [13]. Four days after rootlet emergence, seedlings were transplanted to 500 cm^3^ pots containing Mollisols type soil (USDA classification) and cultivated in a pollinator-free greenhouse under the following regime: 14 to 15 hours of photoperiod with a photosynthetic flux density (PPFD) from 600 to 900 μmol m^-2^ s^-1^ and average temperature of 24°C (Argentinean conditions).

Flowers of *L. tenuis* plants to be cross-pollinated were emasculated and crossed with pollen recovered from *L. corniculatus* using standard techniques. A reciprocal cross using *L. tenuis* as pollen donor and emasculated *L. corniculatus* plants as maternal parent was also performed. Seeds harvested from manually cross-pollinated *L. tenuis* or *L. corniculatus* plants were sown as reported above. Resulting seedlings were transplanted into 300 cm^3^ pots containing perlite/sand (1/1 v/v) and cultivated in a growth chamber under the following regime: 16 hours of white light (PPFD of 450 μmol m^-2^ s^-1^) at 24°C followed by 8 hours in the dark at 20°C and relative humidity ranging from 55 to 65%. All plants were irrigated with Hoagland 0.5 × nutritional solution for 30 days. From an F1 population consisting of 50 *L. tenuis* x *L. corniculatus* plants, four plants representative of one cross (named LH1, LH2, LH3 and LH4) were selected for the further analyses. To this end, each individual hybrid and parents were propagated from cuttings [53]. Kariological analysis was carried out according to Escaray *et al*. [13].

Finally, plants of the F1 hybrid population were randomly intercrossed by manually pollinating the emasculated flowers. An F2 population comprising 200 plants was constituted by sowing seeds recovered and cultivated in a pollinator-free greenhouse, under the conditions described above. Leaves from these plants were harvested for PA staining.

### Morphological and nutritional evaluation of hybrid plants

Clones of hybrid plants were grown simultaneously with clones of parental plants in 2000 cm^3^ pots containing Natracuol typical soil (USDA classification) in a greenhouse under the Argentinian condition described above. Total biomass production was measured over four harvests by growing plants for four months in spring and summer (2010) following a completely randomized experimental design, with ten repetitions per plant. Each month the shoots were cut 3 cm above soil level; the collected material was dried at 37°C and total dry weight determined. Just before the first and second cutting, plants underwent morphological determinations. Stem length and the number of stems per plant were measured and leaf morphology analyzed. Total leaf area, leaf length/width ratio and the number of trichomes/cm^2^ per leaf were determined for the four fully expanded leaves on the main stem. Leaves were scanned and analyzed by Image-Pro Plus 4.5 (Media Cybernetic, Sylver Spring, USA) to measure their area. Trichome number on the adaxial side of leaves was counted under an Optical Microscope (Nikon SMZ800, Nikon, Tokyo) at 10× magnification.

The evaluation of total protein (TP) content and *in vitro* dry matter digestibility (IVDMD) were carried out using dried shoots from *L. corniculatus*, *L. tenuis* and four hybrid plants. Determination of TP was made following the Kjeldahl method [54]. The IVDMD analysis was performed according to Vogel *et al*. [55] on 0.5 g of dried material using a nylon bag with standard porosity incubated in a Daisy II (Ankom) incubator for 48 hours in the presence of the rumen liquor obtained from fistulated cows fed with alfalfa.

### Anthocyanin and chlorophyll determination

To quantify pigment concentrations, clones of the four selected hybrids and of both parents were cultivated for 35 days in a growth chamber, under a completely randomized experimental design with six repetitions per genotype, as reported above.

Anthocyanins were extracted from 100 mg of frozen and ground stem matter with 3 ml of 0.1% HCl/methanol solution for 60 min at room temperature. Then, 0.75 ml of water and 2 ml of chloroform were added to 1 ml of extract. Finally, anthocyanins were quantified by reading the absorbance of the upper phase at 536 nm, and their concentration calculated on the basis of the absorbance of cyanidin-3-0-glucoside. Chlorophyll determination was carried out according to Lichtenthaler [56].

### Proanthocyanidin analysis

Parental and selected F1 plants were cultivated for 35 days in growth chambers, as reported above, and subsequently plant matter was collected and dried at 32°C for 15 days. A completely randomized experimental design with six repetitions per genotype was performed. Proanthocyanidins were quantified as described by Escaray *et al*. [13] following the DMACA-HCl protocol [6].

Levels of PAs were evaluated in the F2 population in the second fully developed leaves by DMACA staining [6]. According to the number of PA-containing cells, related to the intensity of DMACA staining, all 200 plants were sorted into 5 classes. These classes ranged from Class 1, grouping plants with the lowest number of PA-containing cells, to Class 5, clustering plants whose leaf blade was fully covered with PA-containing cells. Then, six F2 plants, three belonging to Class 5 (27, 120, 186) and three to Class 1 (3, 5, 147) were propagated by cuttings and grown in parallel with the original parental lines for 30 days under outdoor conditions in summer 2012. Conditions employed were: 13.5 to 14 hours of photoperiod with a PPFD from 1000 to 1350 μmol m^-2^ s^-1^ and average temperature of 22.5°C (Italian conditions). Proanthocyanidins were quantified and expression levels of the PA structural and regulatory genes were analyzed using a completely randomized experimental design, with three repetitions per genotype.

Qualitative analyses of PAs from parental and F1, as well as from reference species *L. uliginosus*, were performed by Thin Layer Chromatography (TLC) using cellulose plates according to Nybom [57]. Commercial Cyanidin and Delphynidin (Sigma-Aldrich, St. Louis, MO, USA) and leaf PAs from *L. uliginosus* were used as standards.

### DNA and RNA isolation, cDNA synthesis and ITS amplification of DNA and cDNA

DNA was isolated from leaves according to Doyle and Doyle [58]. RNA was isolated from leaves and stems using the Total RNA Plant Isolation kit (Sigma) according to the supplier’s instructions, after which a further DNase treatment was added. The quality and quantity of RNA were verified by agarose gel electrophoresis and spectrophotometric analysis. The absence of DNA from the RNA samples was tested by the null PCR amplification of the universal rDNA primer pair ITS1/ITS4, as described in Paolocci *et al*. [59]. The same primer pair was also used to prove the hybrid status of F1 plants by amplifying the genomic DNA from parental and hybrid lines and sequencing the resulting amplicons. Then cDNA from parental, hybrid F1 and F2 plants was synthesized from 3 μg of total RNA, using SuperScript III H-Reverse Transcriptase (Invitrogen) and 100 pmol of random hexamers (Pharmacia Biotech) according to supplier’s instructions.

### Primer design and cloning of housekeeping and PA genes

The cDNA sequences related to the alpha elongation factor (*EF*-*1α*), to the structural genes of the flavonoid pathway (*PAL*, *CHS*, *DFR*, *ANS*, *ANR*, *LAR1* and *LAR2*) and to regulatory *MYB* and *bHLH* genes from *Lotus* spp. and other model species were downloaded from GenBank. Sequence alignment for each target gene was carried out using the BioEdit software [60]. Then a primer pair specific to each target gene was designed, with the help of OligoExpress Software (Applera Biosystems), on the most conserved nucleotidic residues between species to amplify cDNA isolated from leaves of *L. tenuis* and *L. corniculatus* parental lines, obtained as reported above. The primer pairs are given in Additional file 1: Table S7. The PCR conditions were those reported in Paolocci *et al*. [31]. The resulting amplicons were sequenced, analyzed and incorporated to GenBank (Additional file 1: Table S5). Later were aligned to design gene-specific primer pairs on highly conserved residues between the two parental lines for qRT-PCR analysis (Additional file 1: Table S8).

### Quantitative RT-PCR analysis

The primer pairs, designed as reported above, were initially checked for their specificity and amplification efficiency in qRT-PCR experiments on leaf cDNA from both parents. To test for specificity a dissociation analysis was performed after each amplification run, followed by amplicon sequencing. Meanwhile, PCR efficiency was assessed by performing a standard curve for each gene using six dilution points, each one replicated four times. Unless specifically stated, only primer pairs that produced the expected amplicon and showed similar PCR efficiency on both parents were used.

To study gene expression, the parents and the selected F1 hybrid plants were grown under growth chamber conditions for 30 days as described above. A completely randomized experimental design with four biological replicates for each genotype was used. For each RNA sample, two RT steps were performed and then pooled. An aliquot of 5 μL of 1:10 diluted pools of cDNA was used in the PCR reaction, which was made up using the Power Sybr-Green PCR core mix (Applied Biosystems) according to the supplier’s instructions in 20 μL of final volume in the presence of 2.5 pmol of each primer. Four biological replicates were performed per sample and gene. Cycling parameters were two initial steps of 50°C for 2 min and 95°C for 2 min, a two-step cycle of 95°C for 15 s and 60°C for 1 min repeated 50 times, a final step of 10 min at 60°C. Afterward, the dissociation protocol was performed. Amplifications were performed on ABI PRISM 5700 SDS apparatus (Applied Biosystems). For each transcript, the average threshold cycle (Ct) was determined. The gene quantification method based on the relative expression of the target gene versus the reference genes *EF*-*1α*, was adopted according to Paolocci *et al*. [31].

To study gene expression on the F2 population, selected plants were analyzed in parallel with *L. corniculatus* and *L. tenuis* parental lines. To this purpose, a completely randomized experimental design with three biological replicates per genotype was used and grown under Italian conditions described above. The qRT-PCR analyses were performed following the procedure reported above.

### Sequence analysis

Double-strand sequence analysis was carried out, either directly on PCR products or on PCR fragments previously cloned in the pGEM-T Easy Vector System I (Promega), using the Big Dye Terminator Cycle Sequencing Kit and an ABI Prism 310 Sequence Analyzer (Applied Biosystems) according to the supplier’s instructions. The sequencing primers were the Sp6 and T7 vector primers for cloned PCR fragments and gene-specific primers for direct sequencing reactions.

Sequence data from this article can be found in the GenBank NCBI data libraries under accession numbers [GenBank: KF164612] (LtITS), [GenBank: KF164611] (LcITS), [GenBank: KF134531] (Lt1αEF cDNA), [GenBank: KF134524] (Lc1αEF cDNA), [GenBank: KF428722] (LtbHLH cDNA), [GenBank: KF428721] (LcbHLH), [GenBank: KF134528] (LcTT2 cDNA), [GenBank: KF134534] (LtPAL cDNA), [GenBank: KF134527] (LcPAL cDNA), [GenBank: KF428720] (LtDFR genomic), [GenBank: KF428719] (LcDFR genomic), [GenBank: KF134530] (LtCHS cDNA), [GenBank: KF134523] (LcCHS cDNA), [GenBank: KF134522] (LcANS cDNA), [GenBank: KF134529] (LtANR cDNA), [GenBank: KF134521] (LcANR cDNA), [GenBank: KF134532] (LtLAR1 cDNA), [GenBank: KF134525] (LcLAR1 cDNA), [GenBank: KF134533] (LtLAR2 cDNA), [GenBank: KF134526] (LcLAR2 cDNA), [GenBank: KF386027] (LtLAR2 genomic) and [GenBank: KF386026] (LcLAR2 genomic).

### Statistical analysis

Statistical analysis was performed using the Infostat program [61]. One-way ANOVA was carried out for all analyses and a Tukey test was used for a multivariate analysis (p < 0.05). For non-parametric data the Kruskal-Wallis test with paired comparison (p < 0.05) was performed. The statistical analysis of the relative gene expression was performed according to Pfaffl *et al*. [62].

## Abbreviations

PAs: Proanthocyanidins; ITS: Nuclear ribosomal internal transcriptional spacer; IVDMD: *In vitro* dry matter digestibility; TP: Total protein.

## Competing interests

The authors declare that they have no competing interests.

## Authors’ contributions

FE recovered and propagated the wild diploid Lotus corniculatus plants. FE and MFB performed the crosses and all morphological analyses. FE, VP, FD and FP cloned the genes and performed all molecular analyses. FE, FM, FLP performed quantification of anthocyainins, proanthocyanidins and chlorophylls. FE performed statistical analyses. FE, PC, FP and OAR conceived and designed the experiments. FE, OAR and FP wrote the paper. All authors read and approved the final version of the manuscript.

## Supplementary Material

Additional file 1: Table S1 Characteristics of the plants belonging to the population from Devesa de El Saler. **Table S2.** Main morphological differences between the wild diploid *L. corniculatus* population recovered in Spain and phylogenetically related *Lotus* spp. **Table S3.** Foliar area (expressed in cm^2^) and length/width ratio in central leaflet of *Lotus* parental plants and F1 hybrids. Within each grey block means with a similar letter do not differ significantly (p < 0.05). **Table S4.** Dry weight (g) and leaf/stem weight ratio in *Lotus* parental plants and F1 hybrids. Within each grey block means with a similar letter are not significantly different (p < 0.05). **Table S5.** Length and sequence identity of the amplified genes. **Table S6.** Relative expression of PA genes in leaves of parental plants and F2 selected hybrids with the highest (A27, A120 and A186) and the lowest (B3, B5 and B147) PA levels. Expression was determined by real-time RT-PCR analysis using EF-1α as the housekeeping gene. *L. tenuis* was arbitrarily selected as reference. Means with a similar letter do not differ significantly (p < 0.05). **Table S7.** List of primers used for cloning genes in *Lotus* parental plants. **Table S8.** List of primers used for qRT-PCR analysis. Click here for file

Additional file 2: Figure S1 Stained mitotic meristematic root cells. **Figure S2.** Phylogenetic tree constructed using the Maximum Parsimony method based on ITS sequences. **Figure S3.** Partial alignment of ITS sequences from hybrids and parental plants. **Figure S4.** Chlorophyll and anthocyanin total levels of hybrids and parental plants. **Figure S5.** TLC analysis of anthocyanidins relased by butanol:HCl hydrolysis of PAs from *Lotus* spp. **Figure S6.** Phenotypic classification of the 200 plants of the F2 population according to the PA accumulation patterns. **Figure S7.** Hybrid and parental plants under saline treatment. Click here for file
