# Supplementary material for: Lotus tenuis x L. corniculatus interspecific hybridization as a means to breed bloat-safe pastures and gain insight into the genetic control of proanthocyanidin biosynthesis in legumes
Source: BMC Plant Biol. 2014 Feb 3;14:40. doi: 10.1186/1471-2229-14-40 (PMC3927264; doi:10.1186/1471-2229-14-40)
Supplement: Additional file 1: Table S1 — Characteristics of the plants belonging to the population from Devesa de El Saler. Table S2. Main morphological differences between the wild diploid L. corniculatus population recovered in Spain and phylogenetically related Lotus spp. Table S3. Foliar area (expressed in cm2) and length/width ratio in central leaflet of Lotus parental plants and F1 hybrids. Within each grey block means with a similar letter do not differ significantly (p < 0.05). Table S4. Dry weight (g) and leaf/stem weight ratio in Lotus parental plants and F1 hybrids. Within each grey block means with a similar letter are not significantly different (p < 0.05). Table S5. Length and sequence identity of the amplified genes. Table S6. Relative expression of PA genes in leaves of parental plants and F2 selected hybrids with the highest (A27, A120 and A186) and the lowest (B3, B5 and B147) PA levels. Expression was determined by real-time RT-PCR analysis using EF-1α as the housekeeping gene. L. tenuis was arbitrarily selected as reference. Means with a similar letter do not differ significantly (p < 0.05). Table S7. List of primers used for cloning genes in Lotus parental plants. Table S8. List of primers used for qRT-PCR analysis. [file 1471-2229-14-40-S1.doc]

**Title:** *Lotus tenuis* x *L. corniculatus* interspecific hybridization as a means to breed bloat-safe pastures and gain insight into the genetic control of proanthocyanidin biosynthesis in legumes

**Authors:** Escaray F.J., Passeri V., Babuin M.F., Marco F., Carrasco P., Damiani F., Pieckenstain F.L., Paolocci F. and Ruiz O.A.

Additional file 1: Table S1. Characteristics of the plants belonging to populations from Devesa de El Saler.

|  |  |  |
| --- | --- | --- |
|  |  | **Characteristics** |
|  |  |  |
| **Plants** |  | The plants are sub-glabrous perennial herbs. |
|  |  |  |
| **Leaves** |  | Leaves are 5-foliate and leaflets have ovate-lanceolate form and less than 3 times longer than wide. |
|  |  |  |
| **Inflorescences** |  | Inflorescences have 2 to 7 flowers and trifoliate bract; the flowers pedicels are shorter than calyx tube. The calyx is actinomorphic and obconic, calyx teeth all equal, sub-glabrous, as long as the calyx tube and acute. Corolla is yellow, with purple or red banner veins, banner with the blade longer than the claw, broadly ovate; wings with obovate blade; keel falcate with curved beak. Style is cylindrical, without tooth. |
|  |  |  |
| **Fruit and seeds** |  | Fruit is cylindrical, more or less straight, erect or patent, with 5 to 15 seeds, uniseriate. Seeds are more or less globose, brown or reddish brown. |
|  |  |  |

Additional file 1: Table S2. Main morphological differences between the wild diploid *L. corniculatus* population recovered in Spain and phylogenetically related *Lotus* spp.

| ***L. uliginosus*** |  | ***L. tenuis*** |  | ***L. alpinus*** |  | ***L. corniculatus*** |  | ***L. delortii*** |  | ***L. corniculatus* (Spain)** |
| --- | --- | --- | --- | --- | --- | --- | --- | --- | --- | --- |
|  |  |  |  |  |  |  |  |  |  |  |
| 2n = 12 |  | 2n = 12 |  | 2n = 12 or 24 |  | 2n = 24 |  | 2n = 12 |  | 2n = 12 |
|  |  |  |  |  |  |  |  |  |  |  |
| Stoloniferous plant |  | Plants without stolons | | | | | | |  | Stoloniferous plant |
|  |  |  |  |  |  |  |  |  |  |  |
| Fistulous stems |  | Solid stems | | | | | | |  | Solid stems |
|  |  |  |  |  |  |  |  |  |  |  |
| Leaflets with marked nerves, glaucous |  | Leaflets without marked nerves | | | | | | |  | Leaflets without marked nerves |
|  |  |  |  |  |  |  |  |  |  |  |
| Inflorescences with 5-18 flowers |  | Inflorescences with 1-7 flowers | | | | | | |  | Inflorescences with 1-7 flowers |
|  |  |  |  |  |  |  |  |  |  |  |
|  |  | Superior leaflets are generally 4-5 times longer than wide |  | Superior leaflets are generally less than 3 times longer than wide | | | | |  | Superior leaflets are generally less than 3 times longer than wide |
|  |  |  |  |  |  |  |  |  |  |  |
|  |  | Fruit ± pendulum |  | Fruit ± erect | | | | |  | Fruit ± erect |
|  |  |  |  |  |  |  |  |  |  |  |
|  |  |  |  | Actinomorphic calyx, with all teeth equal | | | | |  | Actinomorphic calyx, with all teeth equal |
|  |  |  |  |  |  |  |  |  |  |  |
|  |  |  |  | Calyx teeth up to 1.5 times longer than tube, triangular and acute | | | | |  | Calyx teeth up to 1.5 times longesr than tube, triangular and acute |
|  |  |  |  |  |  |  |  |  |  |  |
|  |  |  |  | Stem up to 10 cm; leaves with 4.5-7 mm superior leaflets; corolla of 11-16 mm |  | Stem of 20-35 cm; leaves with 6-11 mm superior leflets; corolla of 9-18 mm | | |  | Stem up to 90 cm; leaves with 6-11 mm superior leflets; corolla of 9-18 mm |
|  |  |  |  |  |  |  |  |  |  |  |
|  |  |  |  |  |  | Calyx with 1.5-2.5 mm tube, obconic, relatively narrow; calyx tooth of 1.8-3.5 mm, shorter or even 1.5 times than tube length |  | Calyx with 2.5-4 mm tube, ± cylindrical, relatively wide; calyx tooth of 3-5.5 mm, as long or even 1.5 times than tube length |  | Calyx with 1.5-2.5 mm tube, obconic, relatively narrow; calyx tooth of 1.8-3.5 mm, shorter or even 1.5 times than tube length |
|  |  |  |  |  |  |  |  |  |  |  |

Additional file 1: Table S3. Foliar area (expressed in cm^2^) and length/width ratio in central leaflet of *Lotus* parental plants and F1 hybrids. Within each grey block means with a similar letter do not differ significantly (p<0.05).

|  |  |  |  |  |  |  |  |  |  |  |  |  |  |  |  |  |  |  |  |  |  |
| --- | --- | --- | --- | --- | --- | --- | --- | --- | --- | --- | --- | --- | --- | --- | --- | --- | --- | --- | --- | --- | --- |
| **Genotype** |  | **Total leave** | | | |  | **Trifoliate leaflets** | | | |  | **Basal leaflets** | | | |  | **Length/width ratio** | | | | |
|  |  | **mean** |  | **s.d.** |  |  | **mean** |  | **s.d.** |  |  | **mean** |  | **s.d.** |  |  | **mean** |  | **s.d.** | **Ranks** |  |
|  |  |  |  |  |  |  |  |  |  |  |  |  |  |  |  |  |  |  |  |  |  |
| ***L. corniculatus*** |  | 1.83 | ± | 0.42 | b |  | 1.23 | ± | 0.27 | a |  | 0.59 | ± | 0.15 | c |  | 1.85 | ± | 0.20 | 18.9 | a |
| **LH1** |  | 1.64 | ± | 0.18 | ab |  | 1.25 | ± | 0.15 | a |  | 0.41 | ± | 0.12 | b |  | 1.76 | ± | 0.08 | 11.4 | a |
| **LH2** |  | 1.50 | ± | 0.21 | a |  | 1.20 | ± | 0.25 | a |  | 0.40 | ± | 0.09 | ab |  | 1.90 | ± | 0.13 | 23.1 | ab |
| **LH3** |  | 1.64 | ± | 0.28 | ab |  | 1.31 | ± | 0.32 | a |  | 0.45 | ± | 0.11 | b |  | 2.08 | ± | 0.12 | 40.9 | c |
| **LH4** |  | 1.60 | ± | 0.23 | ab |  | 1.28 | ± | 0.13 | a |  | 0.45 | ± | 0.09 | b |  | 2.02 | ± | 0.10 | 36.2 | bc |
| ***L. tenuis*** |  | 1.50 | ± | 0.28 | a |  | 1.20 | ± | 0.22 | a |  | 0.30 | ± | 0.07 | a |  | 2.26 | ± | 0.14 | 51.67 | c |
|  |  |  |  |  |  |  |  |  |  |  |  |  |  |  |  |  |  |  |  |  |  |

Additional file 1: Table S4. Dry weight (g) and leaf/stem weight ratio in *Lotus* parental plants and F1 hybrids. Within each grey block means with a similar letter are not significantly different (p<0.05).

|  |  |  |  |  |  |  |  |  |  |  |  |  |  |  |  |  |  |  |  |  |
| --- | --- | --- | --- | --- | --- | --- | --- | --- | --- | --- | --- | --- | --- | --- | --- | --- | --- | --- | --- | --- |
| **Genotype** |  | **Leaves** | | | |  | **Stems** | | | |  | **Shoots** | | | |  | **Leaves / stems** | | | |
|  |  | **(mean ± s.d.)** | | | |  | **(mean ± s.d.)** | | | |  | **(mean ± s.d.)** | | | |  | **(mean ± s.d.)** | | | |
|  |  |  |  |  |  |  |  |  |  |  |  |  |  |  |  |  |  |  |  |  |
| ***L. corniculatus*** |  | 15.53 | ± | 2.33 | ab |  | 7.03 | ± | 1.36 | c |  | 22.56 | ± | 3.56 | a |  | 2.23 | ± | 0.23 | a |
| **LH1** |  | 15.18 | ± | 4.00 | ab |  | 14.36 | ± | 4.10 | ab |  | 29.54 | ± | 7.94 | b |  | 1.07 | ± | 0.13 | bc |
| **LH2** |  | 19.43 | ± | 2.71 | a |  | 16.36 | ± | 3.04 | a |  | 35.79 | ± | 5.62 | b |  | 1.20 | ± | 0.10 | b |
| **LH3** |  | 16.38 | ± | 3.42 | a |  | 15.28 | ± | 3.56 | ab |  | 31.66 | ± | 6.83 | b |  | 1.08 | ± | 0.11 | bc |
| **LH4** |  | 11.39 | ± | 3.25 | b |  | 11.60 | ± | 3.70 | bc |  | 22.99 | ± | 6.86 | a |  | 0.99 | ± | 0.11 | c |
| ***L. tenuis*** |  | 18.61 | ± | 5.07 | a |  | 15.08 | ± | 4.44 | ab |  | 33.69 | ± | 9.30 | b |  | 1.25 | ± | 0.15 | b |
|  |  |  |  |  |  |  |  |  |  |  |  |  |  |  |  |  |  |  |  |  |

Additional file 1: Table S5. Length and sequence identity of the amplified genes.

|  |  |  |  |  |  |  |  |
| --- | --- | --- | --- | --- | --- | --- | --- |
| **Gene Bank ID Number** | **Gene** | **Species** | **Length (bp)** | **Description** | **Shows similitude with:** | **Identity** | **Ref.** |
|  |  |  |  |  |  |  |  |
| ***KF134531*** | ***1αEF*** | ***Lt*** | 676 | cDNA | *L. corniculatus*, elongation factor 1-alpha, mRNA partial sequence [GenBank: AY633710] | 98% | 1 |
| ***KF134524*** | ***1αEF*** | ***Lc*** | 638 | cDNA |  | 99% |  |
|  |  |  |  |  |  |  |  |
| ***KF428722*** | ***FebHLH*** | ***Lt*** | 671 | cDNA | *L. japonicus*, transcriptional factor bHLH (LjTAN1), mRNA [GenBank: AB492285] | 99% | 8 |
| ***KF428721*** | ***FebHLH*** | ***Lc*** | 649 | cDNA |  | 99% |  |
|  |  |  |  |  |  |  |  |
|  | ***FeTT2*** | ***Lt*** | 84 | cDNA | *L. japonicus*, transcriptional factor R2R3-MYB (LjTT2b), mRNA complete sequence  [GenBank: AB300034] | 99% | 8 |
| ***KF134528*** | ***FeTT2*** | ***Lc*** | 712 | cDNA |  | 99% |  |
|  |  |  |  |  |  |  |  |
| ***KF134534*** | ***FePAL*** | ***Lt*** | 355 | cDNA | *L. corniculatus*, phenylalanine ammonia-lyase (PAL), mRNA partial sequence  [GenBank: AY633709] | 99% | 1 |
| ***KF134527*** | ***FePAL*** | ***Lc*** | 468 | cDNA |  | 98% |  |
|  |  |  |  |  |  |  |  |
| ***KF428720*** | ***FeDFR*** | ***Lt*** | 1696 | Genomic DNA | *L. corniculatus*, dihydroflavonol 4-reductase (DFRa) [GeneBank: X97576] | 95% | 3 |
| ***KF428719*** | ***FeDFR*** | ***Lc*** | 1751 | Genomic DNA | *L. corniculatus*, dihydroflavonol 4-reductase (DFR), complete sequence  [GenBank: AY633707] | 93% | 1 |
|  |  |  |  |  |  |  |  |
| ***KF134530*** | ***FeCHS*** | ***Lt*** | 314 | cDNA | *L. corniculatus*, chalcone synthase (CHS), partial sequence [GenBank: AF308143] | 93% | 5 |
| ***KF134523*** | ***FeCHS*** | ***Lc*** | 322 | cDNA |  | 94% | 5 |
|  |  |  |  |  |  |  |  |
|  | ***FeANS*** | ***Lt*** | 1348 | Genomic DNA | *L. corniculatus*, anthocyanin synthase (ANS), partial sequence [GenBank: AY028931] | 99% | 6 |
| ***KF134522*** | ***FeANS*** | ***Lc*** | 325 | cDNA |  | 99% | 6 |
|  | ***FeANS*** | ***Lc*** | 1493 | Genomic DNA |  | 98% | 6 |
|  |  |  |  |  |  |  |  |
| ***KF134529*** | ***FeANR*** | ***Lt*** | 451 | cDNA | *L. corniculatus*, anthocyanidin reductase (ANR1-1), mRNA complete sequence  [GenBank: DQ349108] | 99% | 7 |
| ***KF134521*** | ***FeANR*** | ***Lc*** | 465 | cDNA |  | 99% |  |
|  |  |  |  |  |  |  |  |
| ***KF134532*** | ***FeLAR1*** | ***Lt*** | 623 | cDNA | *L. corniculatus*, leucoanthocyanidin reductase (LAR1-2), mRNA complete sequence  [GenBank: DQ349101] | 99% | 7 |
| ***KF134525*** | ***FeLAR1*** | ***Lc*** | 705 | cDNA |  | 99% |  |
|  |  |  |  |  |  |  |  |
| ***KF134533*** | ***FeLAR2*** | ***Lt*** | 498 | cDNA | *L. corniculatus*, leucoanthocyanidin reductase (LAR2-1), mRNA complete sequence  [GenBank: DQ349104] | 99% | 7 |
| ***KF134526*** | ***FeLAR2*** | ***Lc*** | 498 | cDNA |  | 100% |  |
| ***KF386027*** | ***FeLAR2*** | ***Lt*** | 900 | Genomic DNA | *L. corniculatus*, leucoanthocyanidin reductase (LAR2-2), complete sequence  [GenBank: DQ349107] | 98% |  |
| ***KF386026*** | ***FeLAR2*** | ***Lc*** | 918 | Genomic DNA |  | 89% |  |
|  |  |  |  |  |  |  |  |

References: **1**- Paolocci *et al*. 2005. Journal of Experimental Botany 56: 1093-1103; **2**- Sato *et al*. 2008. DNA Research 15: 227-239; **3**- Bavage *et al*. 1997. Plant Molecular Biology 35: 443-458; **4**- Sakurai *et al*. Unpublished; **5**- Allison and Robbins, Unpublished; **6**- Hughes and Robbins, Unpublished; **7**- Paolocci *et al*. 2007. Plant Physiology 143: 504-516; **8**- Yoshida et al. 2010. Plant and Cell Physiology 51: 912-922.

Additional file 1: Table S6. Relative expression of PA genes in leaves of parental plants and F2 selected hybrids with the highest (A27, A120 and A186) and the lowest (B3, B5 and B147) PA levels. Expression was determined by real-time RT-PCR analysis using EF-1α as the housekeeping gene. *L. tenuis* was arbitrarily selected as reference. Means with a similar letter do not differ significantly (p<0.05).

|  |  |  |  |  |  |  |  |  |  |  |  |  |  |  |  |  |  |  |  |  |
| --- | --- | --- | --- | --- | --- | --- | --- | --- | --- | --- | --- | --- | --- | --- | --- | --- | --- | --- | --- | --- |
| **Sample** |  | **Relative expression *PAL*** | | | |  | **Relative expression *CHS*** | | | |  | **Relative expression *DFR*** | | | |  | **Relative expression *ANS*** | | | |
|  |  | (mean ± s.d.) | | | |  | (mean ± s.d.) | | | |  | (mean ± s.d.) | | | |  | (mean ± s.d.) | | | |
|  |  |  |  |  |  |  |  |  |  |  |  |  |  |  |  |  |  |  |  |  |
| ***Lc*** |  | 0.72 | ± | 0.16 | ab |  | 2.07 | ± | 0.50 | c |  | 19.90 | ± | 3.48 | e |  | 11.65 | ± | 2.62 | C |
| ***A27*** |  | 1.18 | ± | 0.20 | c |  | 1.47 | ± | 0.32 | c |  | 8.70 | ± | 3.22 | cd |  | 10.34 | ± | 4.06 | C |
| ***A120*** |  | 0.68 | ± | 0.11 | a |  | 15.33 | ± | 3.55 | e |  | 20.53 | ± | 3.97 | e |  | 4.66 | ± | 1.01 | B |
| ***A186*** |  | 0.98 | ± | 0.21 | bc |  | 4.13 | ± | 0.16 | d |  | 13.11 | ± | 4.45 | d |  | 9.69 | ± | 3.13 | C |
| ***B3*** |  | 0.83 | ± | 0.07 | b |  | 1.12 | ± | 0.29 | bc |  | 2.62 | ± | 0.23 | b |  | 1.58 | ± | 0.41 | A |
| ***B5*** |  | 1.13 | ± | 0.42 | abc |  | 1.10 | ± | 0.23 | bc |  | 6.87 | ± | 0.74 | c |  | 1.78 | ± | 0.51 | A |
| ***B147*** |  | 0.76 | ± | 0.16 | ab |  | 0.74 | ± | 0.16 | a |  | 2.16 | ± | 0.96 | b |  | 1.50 | ± | 0.73 | A |
| ***Lt*** |  | 1.00 | ± | 0.07 | c |  | 1.01 | ± | 0.17 | b |  | 1.02 | ± | 0.22 | a |  | 1.21 | ± | 0.81 | A |
|  |  |  |  |  |  |  |  |  |  |  |  |  |  |  |  |  |  |  |  |  |
| **Sample** |  | **Relative expression *ANR*** | | | |  | **Relative expression *LAR1*** | | | |  | **Relative expression *LAR2*** | | | |  | **Relative expression *TT2*** | | | |
|  |  | (mean ± s.d.) | | | |  | (mean ± s.d.) | | | |  | (mean ± s.d.) | | | |  | (mean ± s.d.) | | | |
|  |  |  |  |  |  |  |  |  |  |  |  |  |  |  |  |  |  |  |  |  |
| ***Lc*** |  | 9.33 | ± | 2.11 | cd |  | 354.05 | ± | 112.04 | c |  | 17.98 | ± | 8.65 | e |  | 56.59 | ± | 11.54 | D |
| ***A27*** |  | 11.13 | ± | 4.68 | d |  | 276.72 | ± | 79.84 | c |  | 3.81 | ± | 1.50 | cd |  | 55.11 | ± | 16.82 | D |
| ***A120*** |  | 4.57 | ± | 1.62 | c |  | 339.65 | ± | 81.23 | c |  | 9.02 | ± | 4.59 | de |  | 36.75 | ± | 1.76 | d |
| ***A186*** |  | 9.20 | ± | 2.20 | cd |  | 351.57 | ± | 69.98 | c |  | 13.78 | ± | 8.55 | de |  | 47.96 | ± | 8.00 | d |
| ***B3*** |  | 1.25 | ± | 0.17 | a |  | 1.03 | ± | 0.72 | ab |  | 0.13 | ± | 0.22 | a |  | 5.44 | ± | 2.78 | c |
| ***B5*** |  | 1.93 | ± | 0.45 | b |  | 1.98 | ± | 0.79 | b |  | 0.42 | ± | 0.53 | abc |  | 2.31 | ± | 0.82 | b |
| ***B147*** |  | 1.18 | ± | 0.66 | ab |  | 0.67 | ± | 0.67 | a |  | 1.12 | ± | 0.91 | b |  | 3.36 | ± | 1.86 | bc |
| ***Lt*** |  | 1.19 | ± | 0.73 | ab |  | 1.12 | ± | 0.67 | ab |  | 1.12 | ± | 0.66 | b |  | 1.00 | ± | 0.06 | a |
|  |  |  |  |  |  |  |  |  |  |  |  |  |  |  |  |  |  |  |  |  |
| **Sample** |  | **Relative expression *bHLH*** | | | |  | **PA soluble** | | | |  | **PA insoluble** | | | |  | **PA total** | | | |
|  |  | (mean ± s.d.) | | | |  | (mg / g DM, mean ± s.d.) | | | |  | (mg / g DM, mean ± s.d.) | | | |  | (mg / g DM, mean ± s.d.) | | | |
|  |  |  |  |  |  |  |  |  |  |  |  |  |  |  |  |  |  |  |  |  |
| ***Lc*** |  | 0.40 | ± | 0.14 | a |  | 13.30 | ± | 1.18 | d |  | 17.69 | ± | 1.67 | d |  | 31.00 | ± | 0.63 | e |
| ***A27*** |  | 0.93 | ± | 0.22 | c |  | 4.48 | ± | 0.66 | b |  | 8.69 | ± | 1.76 | b |  | 13.17 | ± | 1.12 | b |
| ***A120*** |  | 0.84 | ± | 0.28 | bcd |  | 7.63 | ± | 1.39 | c |  | 12.31 | ± | 0.66 | c |  | 19.94 | ± | 1.96 | c |
| ***A186*** |  | 0.83 | ± | 0.23 | b |  | 7.90 | ± | 0.23 | c |  | 18.63 | ± | 0.86 | d |  | 26.53 | ± | 0.97 | d |
| ***B3*** |  | 0.70 | ± | 0.05 | b |  | 0.02 | ± | 0.03 | a |  | 0.50 | ± | 0.06 | a |  | 0.52 | ± | 0.03 | a |
| ***B5*** |  | 0.62 | ± | 0.11 | b |  | 0.04 | ± | 0.03 | a |  | 0.90 | ± | 0.37 | a |  | 0.93 | ± | 0.40 | a |
| ***B147*** |  | 0.67 | ± | 0.19 | bc |  | 0.02 | ± | 0.01 | a |  | 0.94 | ± | 0.08 | a |  | 0.96 | ± | 0.10 | a |
| ***Lt*** |  | 1.00 | ± | 0.06 | d |  | 0.03 | ± | 0.02 | a |  | 0.62 | ± | 0.17 | a |  | 0.65 | ± | 0.17 | a |
|  |  |  |  |  |  |  |  |  |  |  |  |  |  |  |  |  |  |  |  |  |

Additional file 1: Table S7. List of primers used for cloning genes in *Lotus* parental plants.

|  |  |  |  |  |  |
| --- | --- | --- | --- | --- | --- |
| **Gene name** | **Primer name** | **Sense** | | **Sequence** |  |
|  |  |  |  |  |  |
| ***Elongation factor 1-alpha*** | **FeEfact f1** | Forward | 5´ | ATTGTGGTSATTGGCCAYGTCGA | 3´ |
|  | **FeEfact r1** | Reverse | 5´ | CCAATCTTGTASACATCCTGAAGGG | 3´ |
|  | **FeEfact r2** | Reverse | 5´ | TTGATCTGGTCAAGAGCCTCAAGA | 3´ |
|  |  |  |  |  |  |
| ***Phenylalanine ammonia-lyase*** | **FePAL f1** | Forward | 5´ | GGCTGCACARAAGKTRCATGAGAT | 3´ |
|  | **PAL fw** | Forward | 5´ | GGRCAATGYGGYAACCARATCGG | 3´ |
|  | **FePAL r1** | Rvs | 5´ | GCMCCTTTGAAKCCRTAATCYAARCT | 3´ |
|  | **PAL Rev** | Rvs | 5´ | GCATCYTGGTAYTGYTGGTAYTC | 3´ |
|  |  |  |  |  |  |
| ***Dihydroflavonol 4-reductase*** | **DFR frw1** | Forward | 5´ | CCTGAGAATGAARTRATCAAGCC | 3´ |
|  | **FeDFR f1** | Forward | 5´ | GGTTTCATCGGGTCMTGGCTT | 3´ |
|  | **DFR backw1** | Reverse | 5´ | TAATGAGCYTCRCCAGT | 3´ |
|  | **FeDFR r1** | Reverse | 5´ | GCTCCWGTGTACATATCCTCTAAGSTG | 3´ |
|  | **FeseqDFRr** | Reverse | 5´ | TGAACTATCATTGTACCGGTG | 3´ |
|  |  |  |  |  |  |
| ***Chalcone synthase*** | **FeCHS f1** | Forward | 5´ | CTCCAGATAGKGAVGGAGCCA | 3´ |
|  | **FeCHS f2** | Forward | 5´ | TTYCATYTCCTTAAAGATGTYCHGGG | 3´ |
|  | **FeCHS r1** | Reverse | 5´ | GCTGAYTTCYTTCTCATTTCATCTARGATG | 3´ |
|  | **FeCHS r2** | Reverse | 5´ | GGACACATGCACTTGACATRTTACC | 3´ |
|  |  |  |  |  |  |
| ***Anthocyanin synthase*** | **FeANS f1** | Forward | 5´ | AGCAAGCTKGCMAACAATGC | 3´ |
|  | **FeseqANSf3** | Forward | 5´ | GTCACCCGTTGCATTAC | 3´ |
|  | **FeseqANSf2** | Forward | 5´ | GAAGAATCAATCATGTGTTACC | 3´ |
|  | **FeseqANSf1** | Forward | 5´ | GTAAAATTTGTAACTRATTGTGG | 3´ |
|  | **FeANS r1** | Reverse | 5´ | TCRGTGTGRGCTTCAACWCC | 3´ |
|  | **FeANS r2** | Reverse | 5´ | GGGGCATTTTGGGTAGTAGTTGAT | 3´ |
|  | **FeseqANSr** | Reverse | 5´ | CAATAGAGAAAGGGCACCTCTT | 3´ |
|  |  |  |  |  |  |
| ***Anthocyanidin reductase*** | **FeANR f1** | Forward | 5´ | CCGTATAATAGTGCTCACTTTTCTGAAGTT | 3´ |
|  | **FeseqANRf** | Forward | 5´ | GGCATATTTAGACAACAGAAAC | 3´ |
|  | **FeANR r1** | Reverse | 5´ | TCAGTCCAGTTGCTTTCATCCATAAC | 3´ |
|  | **FeANR r2** | Reverse | 5´ | TGCAGGCTTGATCATGTCATTCTC | 3´ |
|  | **FeseqANRr** | Reverse | 5´ | ATAGGCATTTGGTGGGTA | 3´ |
|  |  |  |  |  |  |
| ***Leucoanthocyanidin reductase 1*** | **FeLAR1 f1** | Forward | 5´ | TCGGAGSAACBGGTTTCATRGG | 3´ |
|  | **FeLAR1 f2** | Forward | 5´ | TCAGTTCRTRACYAAGGCAAG | 3´ |
|  | **FeLAR1 r1** | Reverse | 5´ | GRCAACCDTTGATGAAWATRTCATGVGTG | 3´ |
|  | **FeLAR1 r2** | Reverse | 5´ | CAATTGTGAACTTTCCAATATCAWTGCCATC | 3´ |
|  |  |  |  |  |  |
| ***Leucoanthocyanidin reductase 2*** | **FeLAR2 f1** | Forward | 5´ | CGGAGCAACTGGTTTCATGGG | 3´ |
|  | **FeLAR2 f2** | Forward | 5´ | ATRAATGATAAGGAGTTCATGCAGAAGA | 3´ |
|  | **FeLAR2 r1** | Reverse | 5´ | CAGTGCCATCACCATATATGTGC | 3´ |
|  | **FeLAR2 r2** | Reverse | 5´ | GGGTGGAGGAAGCTGTGATGG | 3´ |
|  |  |  |  |  |  |
| ***R2R3-MYB transcription factor*** | **LcTT2 f1** | Forward | 5´ | CACCGACCCACGACTTAGGCAAAACA | 3´ |
|  | **LcTT2 f2** | Forward | 5´ | CACCGTGAGAGTGAAATGGGAAGAAGCCCT | 3´ |
|  | **LcTT2 r1** | Reverse | 5´ | GGCATTAGATCCTCTTGGTTGTTGTACG | 3´ |
|  | **LcTT2 r2** | Reverse | 5´ | CAATTCAGTTCCCACCCATCCCA | 3´ |
|  | **Lc TT2 rstran** | Reverse | 5´ | AAGAGGAGGAGAAGCAGCACCAA | 3´ |
|  |  |  |  |  |  |

Additional file 1: Table S8. List of primers used for qRT-PCR analysis.

|  |  |  |  |  |  |
| --- | --- | --- | --- | --- | --- |
| **Gene name** | **Primer name** | **Sense** | | **Sequence** |  |
|  |  |  |  |  |  |
| ***R2R3-MYB transcription factor*** | **qFeTT2f** | Forward | 5´ | GCAGGTTTGAAGCGTTGTGG | 3´ |
|  | **qFeTT2r** | Reverse | 5´ | TGATATATTGCCTCTCTTGATACCTGG | 3´ |
|  |  |  |  |  |  |
| ***bHLH transcription factor*** | **qFebHLHf** | Forward | 5´ | CAGAGGATCTCTCAGATTCAGAGTGG | 3´ |
|  | **qFebHLHr** | Reverse | 5´ | CCAGGCAAACTTTGGTTGGG | 3´ |
|  |  |  |  |  |  |
| ***Phenylalanine ammonia-lyase*** | **qFePALf** | Forward | 5´ | GGCACCCCAATTGGTGTTT | 3´ |
|  | **qFePALr** | Reverse | 5´ | CGGTGAACTGAGCAAACATGAG | 3´ |
|  |  |  |  |  |  |
| ***Chalcone synthase*** | **qFeCHSf** | Forward | 5´ | CAATTTTTTGGATTGCACACCC | 3´ |
|  | **qFeCHSr** | Reverse | 5´ | GAATAGGACACATGCACTTGACATG | 3´ |
|  |  |  |  |  |  |
| ***Dihydroflavonol 4-reductase*** | **qFeDFRf** | Forward | 5´ | GTCCACTTGGATGATCTTTGTCTTG | 3´ |
|  | **qFeDFRr** | Reverse | 5´ | AATGTCATGGATAGTAGCCTCAGATG | 3´ |
|  |  |  |  |  |  |
| ***Anthocyanin synthase*** | **qFeANSf** | Forward | 5´ | GGAAGTTACCAGCGACTATGCAA | 3´ |
|  | **qFeANSr** | Reverse | 5´ | CCTTCCTTCTTCGAGACCCAA | 3´ |
|  |  |  |  |  |  |
| ***Anthocyanidin reductase*** | **qFeANRf** | Forward | 5´ | CCAACTCGCTACACCTGTGAATTT | 3´ |
|  | **qFeANRr** | Reverse | 5´ | TGACCCGTTTAACTTTCGCCC | 3´ |
|  |  |  |  |  |  |
| ***Leucoanthocyanidin reductase 1*** | **qFeLAR1f** | Forward | 5´ | GCTAAATCATTCATGGTGTTATAAACGA | 3´ |
|  | **qFeLAR1r** | Reverse | 5´ | ACCTACGAGTGAAATGACAACGTCTAT | 3´ |
|  |  |  |  |  |  |
| ***Leucoanthocyanidin reductase 2*** | **qFeLAR2f** | Forward | 5´ | CCTTCAGAATTTGGGCACGAT | 3´ |
|  | **qFeLAR2r** | Reverse | 5´ | TTCGAACAGATGTAGGTGTATGGGA | 3´ |
|  |  |  |  |  |  |
| ***Elongation factor 1-alpha*** | **qFeEFACTf** | Forward | 5´ | TGACAAGCGTGTGATCGAGAGG | 3´ |
|  | **qFeEFACTr** | Reverse | 5´ | GATACCTCTTTCACGCTCAGCCTT | 3´ |
|  |  |  |  |  |  |
