# Supplementary material for: Lotus tenuis x L. corniculatus interspecific hybridization as a means to breed bloat-safe pastures and gain insight into the genetic control of proanthocyanidin biosynthesis in legumes
Source: BMC Plant Biol. 2014 Feb 3;14:40. doi: 10.1186/1471-2229-14-40 (PMC3927264; doi:10.1186/1471-2229-14-40)

Title

Lotus tenuis x *L. corniculatus* interspecific hybridization as a means to breed bloat-safe pastures and gain insight into the genetic control of proanthocyanidin biosynthesis in legumes

Authors

Escaray F.J., Passeri V., Babuin M.F., Marco F., Carrasco P., Damiani F., Pieckenstain F.L., Paolocci F. and Ruiz O.A.

Figure S1. Stained mitotic meristematic root cells. (a) Diploid *L. corniculatus*. **(b)** Hybrid plant (LH2). **(c)** *L. tenuis*. Bars represent 10 µm.

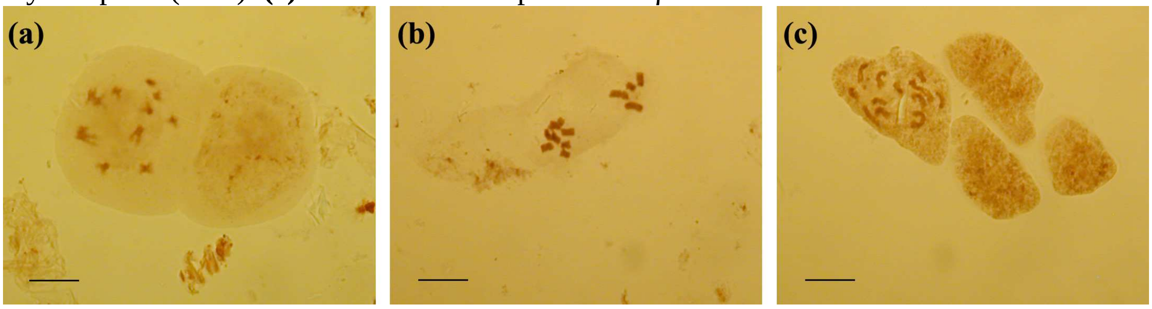

Figure S2. Phylogenetic tree constructed using the Maximum Parsimony method [1] based on ITS sequences. The bootstrap consensus tree inferred from 1000 replicates is taken to represent the evolutionary history of the taxa analyzed [2]. Branches corresponding to partitions reproduced in less than 30% bootstrap replicates are collapsed. The percentage of replicate trees in which the associated taxa clustered together in the bootstrap test (1000 replicates) are shown next to the branches [2]. The MP tree was obtained using the Close-Neighbor-Interchange algorithm [3, pg. 128] with search level 3 [2, 3] in which the initial trees were obtained with the random addition of sequences (10 replicates). Phylogenetic analyses were conducted in MEGA4 [4]. The ITS sequences of *L. tenuis* and diploid *L corniculatus* plants analyzed in this study are given in bold.

1. Eck RV and Dayhoff MO (**1966**). *Atlas of Protein Sequence and Structure*. National Biomedical Research Foundation, Silver Springs, Maryland.

2. Felsenstein J (**1985**). Confidence limits on phylogenies: An approach using the bootstrap. *Evolution* **39**:783-791.

3. Nei M & Kumar S (**2000**). *Molecular Evolution and Phylogenetics*. Oxford University Press, New York.

4. Tamura K, Dudley J, Nei M & Kumar S (**2007**). MEGA4: Molecular Evolutionary Genetics Analysis (MEGA) software version 4.0. *Molecular Biology and Evolution* **24**:1596-1599.

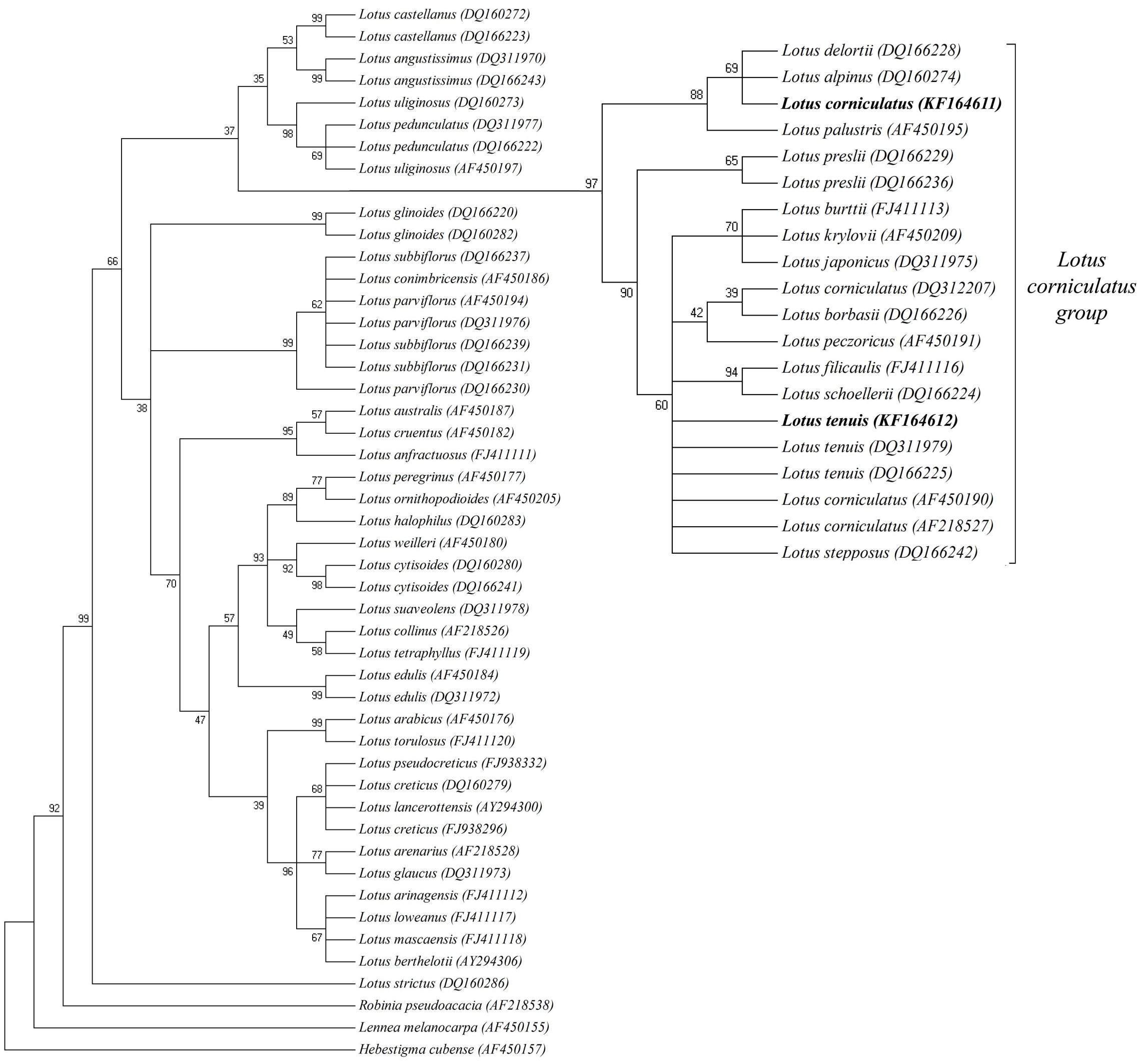

Figure S3. Partial alignment of ITS sequences from hybrid and parental plants. Partial sequences (57 bp) from the parental plants (diploid *L. corniculatus* and *L. tenuis*) and different ITS clones from LH1 and LH3 F1 hybrids.

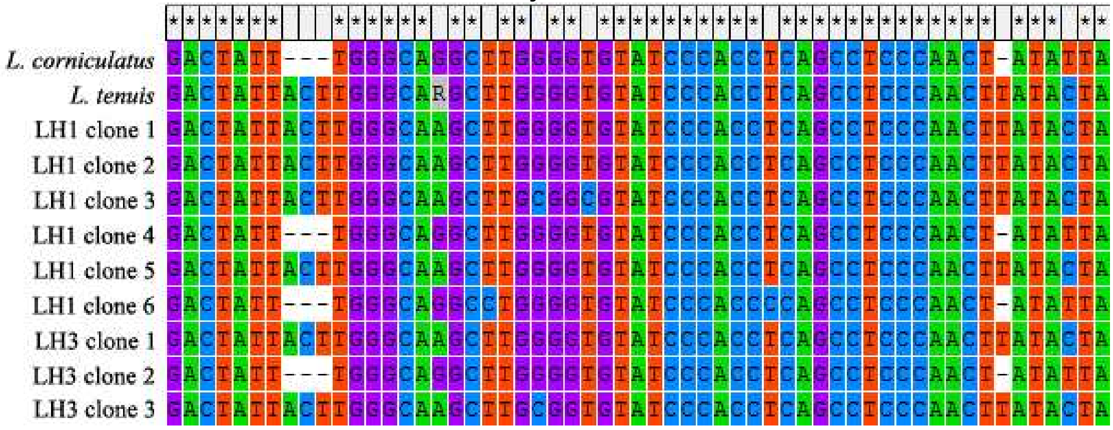

Figure S4. Chlorophyll and anthocyanin total levels of hybrid and parental plants. (a) total chlorophyll content in leaves (mg of total chlorophyll/g of dry matter). **(b)** anthocyanins in stems (mmol cyanidin-3-0-glucoside/g of fresh weight). Samples are as in Fig 2. Bars indicate standard deviation of mean values. Means with a similar letter do not differ significantly ($p<0.05$).

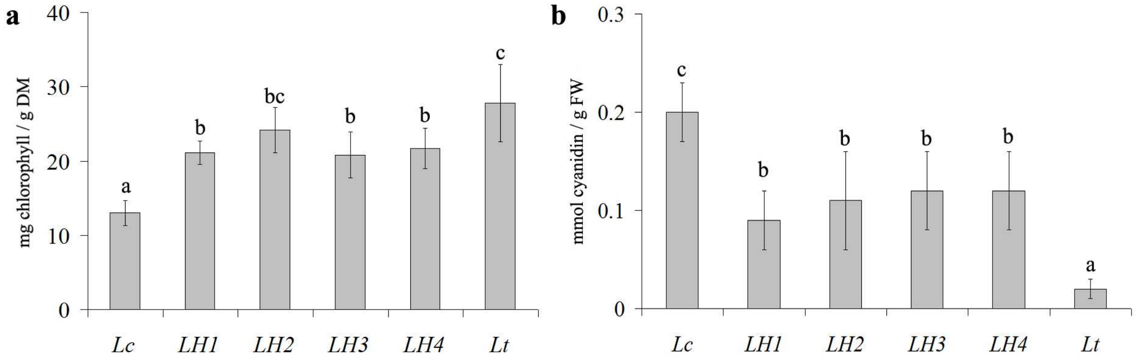

Figure S5. TLC analysis of anthocyanidins relased by butanol:HCl hydrolysis of PAs from *Lotus* spp. Lc: diploid *L. corniculatus*; LH1, LH2, LH3 and LH4: F1 hybrids; Lu: *L. uliginosus*. Dpy and Cy: delphinidin and cyanidin commercial standards, respectively.

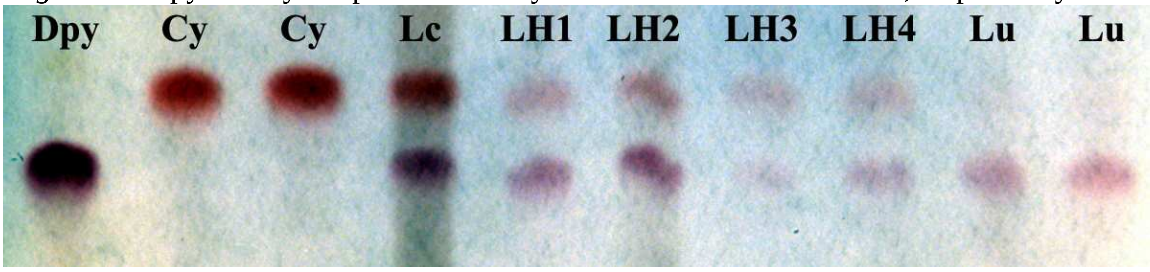

Figure S6. Phenotypic classification of the 200 plants of the F2 population according to the PA accumulation patterns. The PA accumulation pattern for each of the five classes is given at the top of the figure.

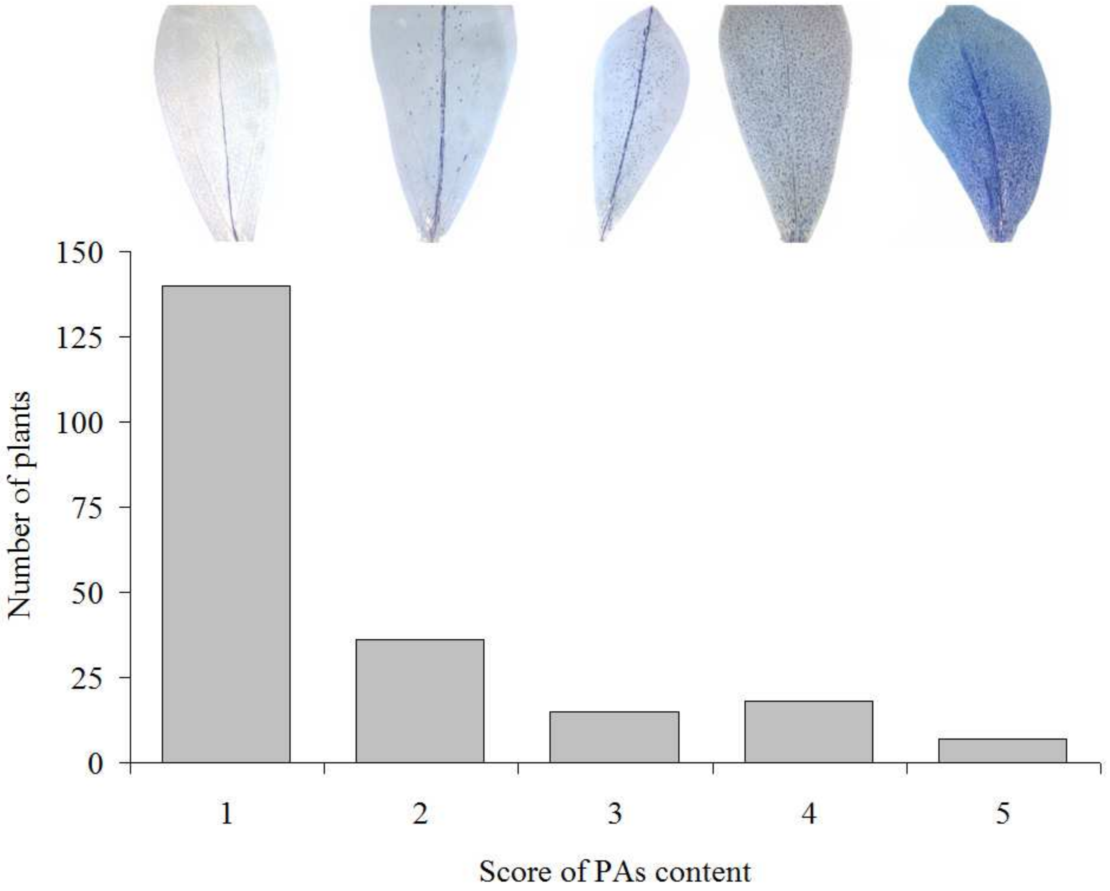

Figure S7. Hybrid and parental plants under saline treatment. Plants of **(a)** *L. tenuis*, **(b)** diploid *L. corniculatus*, **(c)** LH1, **(d)** LH2, **(e)** LH3 and **(f)** LH4. Grown for 45 days under control condition **(control)** and gradual step salt acclimation up to 150 mM of NaCl **(saline)**.

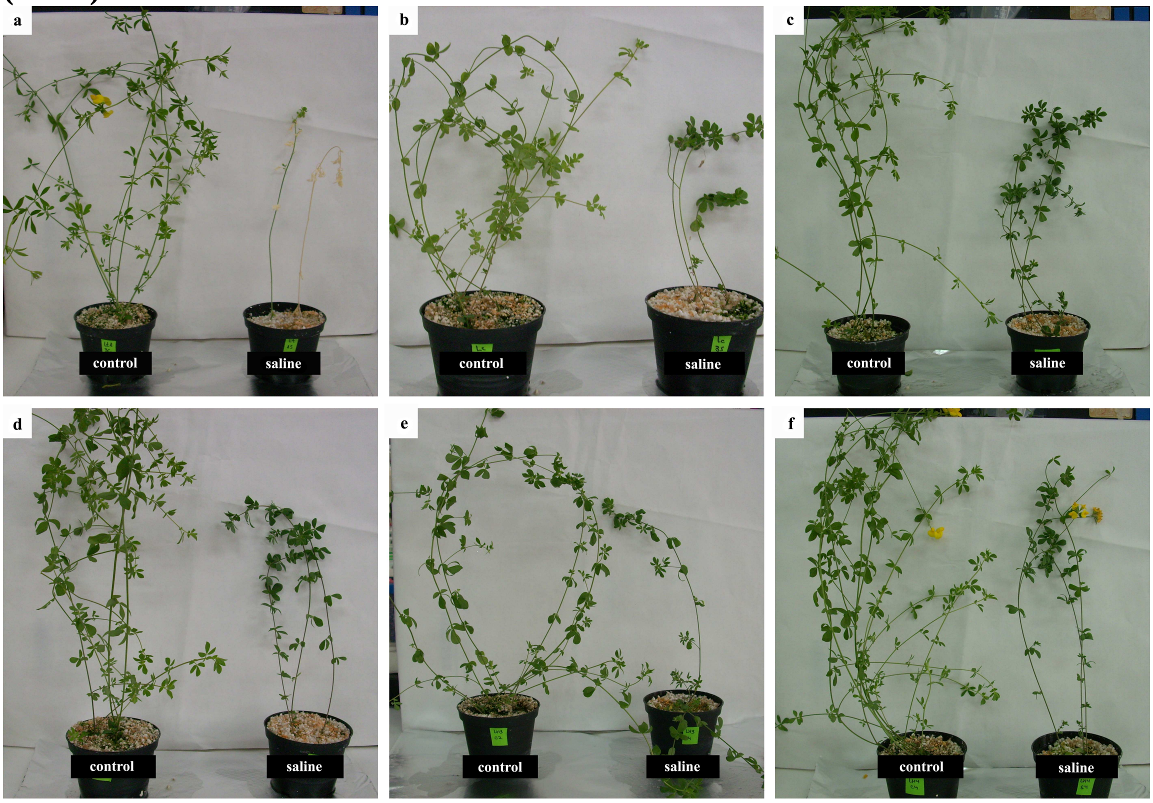

Supplement: Additional file 2: Figure S1 — Stained mitotic meristematic root cells. Figure S2. Phylogenetic tree constructed using the Maximum Parsimony method based on ITS sequences. Figure S3. Partial alignment of ITS sequences from hybrids and parental plants. Figure S4. Chlorophyll and anthocyanin total levels of hybrids and parental plants. Figure S5. TLC analysis of anthocyanidins relased by butanol:HCl hydrolysis of PAs from Lotus spp. Figure S6. Phenotypic classification of the 200 plants of the F2 population according to the PA accumulation patterns. Figure S7. Hybrid and parental plants under saline treatment. [file 1471-2229-14-40-S2.pdf]
